# Adolescent-led *Our Voice* method to promote healthy and active lifestyles and environments in South Africa

**DOI:** 10.1371/journal.pgph.0007141

**Published:** 2026-09-02

**Authors:** Feyisayo A. Wayas, Namhla Matwa, Sacha West, Abby C. King, Tolu Oni, Estelle V. Lambert

**Affiliations:** 1 Research Centre for Health through Physical Activity, Lifestyle & Sport, Division of Physiological Sciences, Department of Human Biology, Faculty of Health Sciences, University of Cape Town, Cape Town, South Africa; 2 IMS Epidemiology, Institute of Metabolic Science, School of Clinical Medicine, University of Cambridge, Cambridge, United Kingdom; 3 Department of Sport Management, Centre for Sport Business and Technology Research (CSBTR), Cape Peninsula University of Technology, Cape Town, South Africa; 4 Department of Epidemiology & Population Health, Stanford University School of Medicine, Stanford, California, United States of America; 5 Department of Medicine, Stanford Prevention Research Center, Stanford University School of Medicine, Stanford, California, United States of America; 6 School of Health, Psychological and Medical Sciences, and Institute for Health Research, University of Southern Queensland, Ipswich, Queensland, Australia; 7 Department of Human Kinetics and Ergonomics, Rhodes University, Makhanda, South Africa; PLOS: Public Library of Science, UNITED STATES OF AMERICA

## Abstract

Adolescents’ immediate environments, including their homes, schools, and neighbourhoods, have a significant impact on their behaviour, health and development. Fostering adolescents’ agency and empowerment can drive environmental and behavioural changes that can lead to improved health outcomes and reduced disparities in current and future generations. The objective of the study was to pilot and evaluate the implementation of a “by the people” citizen science method (the *Our Voice* method), engaging adolescent high school learners in Cape Town, Western Cape, South Africa in promoting healthy and active lifestyles and environments. Sixty-six high school adolescents of both genders between the ages of 13–18 years old were purposively recruited from 6 high schools in Cape Town, located in places of low or middle/high socioeconomic areas (SEA), as citizen scientists. The citizen scientists and some caregivers were interviewed via telephone. Citizen scientists also used a mobile application to collect data in their immediate environments to identify barriers to healthy eating, physical activity, hygiene, and safety. Data were audio-recorded, downloaded, and transcribed. In facilitated workshops, citizen scientists analysed the data and proffered solutions to the barriers, advocated change, and thereafter implemented relevant health promoting changes. Demographic characteristics and anthropometric measurements of the adolescents were also collected. Overall, similar barriers were experienced by adolescents regardless of SEA. However, lower socioeconomic status areas were a significant contributor of barriers to physical inactivity and unhealthy eating, safety, and hygiene. The buy-in and participation of adolescents and the school authority in the intervention to promote healthy lifestyles and environments is imperative. The *Our Voice* method was found to be a promising multi-layered tool for empowering adolescents as advocates in promoting feasible healthy and active lifestyle and environmental changes.

## Introduction

The adolescent stage (10–19 years) is a period of transition accompanied by physical, psychological and social development [1]. The behaviours, lifestyle and exposures experienced from their immediate environments, including their homes, schools, and neighbourhood, during adolescence often have lasting effects on health in adulthood [2]. Notably, approximately 90% of the world's adolescents, who have the highest rates of overweight and lowest rates of physical activity (PA), live in low- and middle-income countries (LMICs) [3,4].These circumstances underscore the importance of addressing adolescent health and development especially in LMICs.

Fostering adolescent collaboration, agency and empowerment, can drive environmental and behavioural changes that can lead to health promotion, improved health outcomes and reduced disparities in the current and future generation [5,6]. Several systematic reviews and studies have highlighted the importance of participatory action research (action research emphasising participation and action by members of communities such as adolescents affected by that research) in tackling context-specific socioecological challenges and disparities of healthy and active lifestyles [5–7]. Importantly, participatory action research embeds learning and change in its process and outcomes [8,9] and can be used to promote multi-component school-based health-promoting interventions which have proven to be effective [10,11]. The school setting plays an important role in health promotion in children and adolescents [12], supported by WHO’s Health Promoting Schools (HPS) framework in which schools are positioned as a healthy setting for living, learning and working to strengthen health promotion and for adolescents to be active agents of change. This is with the expected outcomes of improved learners’ health and well-being, enhanced educational outcomes, reduced health inequalities and creating safe, supportive, and inclusive school environments [13].

Participatory action research, especially those grounded in a systems and/or socioecological framework are very limited in LMICs, especially in Africa. A death gap identified in LMICs research include the insufficient availability of systematic, evidence-based and scalable participatory methods that engage and empower community members such as adolescents who are mostly impacted by the challenges [14–16].

Approximately 60% of adolescents in South Africa currently live in poverty, [17], and only about half (51.7%) of the South African adolescents cohort of both genders met the minimum recommended weekly PA of 420 minutes weekly [18] with most having unhealthy diet food and preferences [19]. Furthermore, the prevalence of adverse lifestyle behaviours, including physical inactivity, overweight/obesity are all higher in the Western Cape than the rest of South Africa. Thus, there is a strong case for NCD prevention in South Africa, particularly, the Western Cape, especially from the adolescent stage, Life orientation (LO), mandatory subject in South African high schools, aligns with the HSP framework by providing structured health education within the classroom, aimed to promote learners’ wellbeing [20].

This study aims to use a type of participatory action (citizen science) research (involving people who are impacted by the research) called the **“**by the people” citizen science also referred to as *Our Voice* method. This method empowers communities participating directly in collecting relevant information about their local contexts that often impacts them, collectively analysing and prioritizing their information, and then developing and collaborating with relevant partners for relevant actions or interventions [21,22].

The *Our Voice* method builds on photovoice theories and best practices as participatory action-based citizen science to support community-driven data collection, analysis, and advocacy [23]. The method uses a 4-step engagement process (Discover, Discuss, Advocate and Change the issue/challenge) has been used extensively conducted in both LMICS and high-income countries (HICs) across various age groups, but with limited studies in African countries. A growing number of *Our Voice* citizen science youth research projects have been conducted in both LMICs and HICs in recent years, with outstanding results [15,24–26] An overview of 20 *Our Voice* studies with youth and young adults (≤24 years old) across 9 countries, primarily HICs and non- African LMICS, highlighted the multi-component health promotion characteristics of the method and that youth and young adults regardless of their socioeconomic status or context specific issues can be involved and empowered to participate in research and make positive change [15]. This is the first adolescent *Our Voice* study conducted in Africa with a focus on the multiple challenges of diet, PA, safety and hygiene.

The overarching aim of this project was to pilot and evaluate the implementation of the *Our Voice* method. The study engaged adolescent learners (attending high schools situated in both lower and higher-income communities) in Cape Town, Western Cape, South Africa to identify and advocate for changes in the school, community or home environments, and to address barriers to healthy eating, PA, safety and hygiene. This study aligns with the World Health Organization Health Promoting Schools (HPS) Framework by engaging adolescents as active change agents in the co-creation of health initiatives within schools and other immediate environments. The expected outcomes will ultimately be to co-create strategies and solutions to address their barriers.

## Materials and methods

### Study design and Settings

The study was conducted in high schools in Cape Town Western Province, South Africa with adolescents between the ages of 13–18 years old. The study is an extension of a work package within a larger study by a research network, Global Diet and Activity Research Network (GDAR) [27,28].The study utilized a predefined sample of between 10–15 adolescents in each school which in qualitative research is adequate for our methodological approach (*Our Voice* method) to support a rigorous analysis and generate meaningful insights relevant to the research questions [23]. The larger study work package focused on understanding the socioecological levers of diet and PA of adolescents.. More information about the research network and the work package can be found elsewhere [27,28].

Data for this study were collected between April 2019 and May 2022. Six schools, four located in areas of low socioeconomic status (SEA) and two located in middle/high SEA in Cape Town, South Africa were purposively selected to participate in the study. The selected schools were categorised into low and middle/high SEA based on the 2021 Socio-economic Profile: City of Cape Town of the school’s location [29]. Initially three schools (one in a low SEA and two located in middle- high SEA) were selected. Three additional schools located in low-income areas were recruited to allow for a more extensive study and comparison. These schools were selected based on preliminary findings from the initial three schools, which indicated that schools in low-income areas faced greater contextual barriers to promoting and adopting healthy lifestyles among adolescents compared to their counterparts, with hygiene and safety as additional critical barriers.

### Recruitment of adolescents and teacher liaison officers

A representative of the research team met individually with all the school principals of the selected schools to explain the purpose and details of the study. A purposive convenience sampling recruitment strategy was used in this study. In consultation with the school principal, a teacher liaison officer (TLO) was recruited as the school “point” person to provide support for the learners involved in the “citizen science” components and to act as a liaison between the researchers, the school and the learners. Between 10–15 adolescents from Grades 8–11 were purposively recruited from each of the six [6] schools. The adolescents of both genders included recognized peer leaders or self-nominated adolescents without any specific leadership role.

### Qualitative methods: *Our Voice* method and in-depth interviews

The study employed a qualitative participatory action research approach, the *Our Voice* method [15,22], supplemented with descriptive quantitative analysis. The Our *Voice* method engages non-scientists to gather data alongside researchers to foster a healthier community and promote better lifestyles. These non-researchers are called citizen scientists (CS). The primary objective of the *Our Voice* method is to provide users, regardless of education level or cultural background, with an easy-to-use mobile tool and platform that allows them to collectively discover, discuss, activate, and improve factors in their local community to create a healthier place for healthy and active living. In addition, as the first set of data from the initial first three schools was collected during COVID when schools were closed, requiring social distancing and movement restrictions, telephone interviews were also conducted with the adolescents and their caregivers in these schools on the barriers to diet and PA in their home, school and neighbourhood.

### Mobile Application: Discovery Tool and Epicollect 5

The mobile applications that were used for this study to collect data were the Discovery Tool, which is a neighbourhood environmental assessment mobile application developed by the *Our Voice* Global Citizen Science Research Initiative at Stanford University [30], and the Epicollect 5, developed by the Centre for Genomic Pathogen Surveillance as part of the Big Data Institute at Oxford University [31]. We switched to the use of the Discovery Tool from the Epicollect 5 after data collection from 3 schools due to an established relationship with the Discovery Tool platform developer and technical support team, enabling real-time technical support. However, the instructions, prompts and usability of both tools was comparable, which is expected to have minimal impact for data comparability. Both mobile applications can be used on either the Android or Apple iOS, and can collect data such as photos, GPS-based walking route mapping, and geo-tagged audio narratives/voice notes. Both tools have been used in a range of countries [32–35]. The mobile applications do not require internet/data connection when being used; a Wi-Fi or internet connection is only needed when the data is being downloaded.

**Fig 1:**
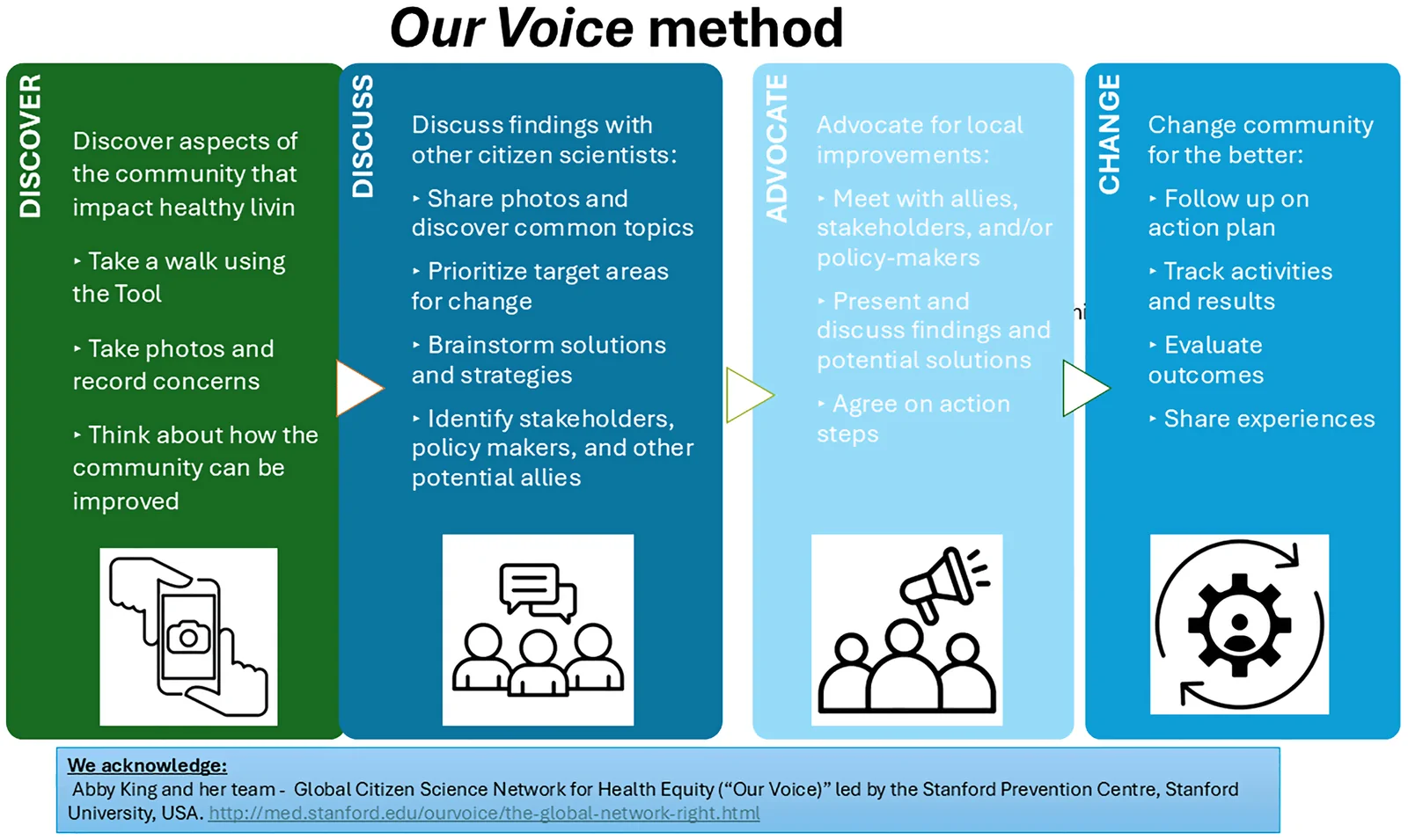
*Our Voice* Method. This figure illustrates the key steps of the *Our Voice* Method.

The *Our Voice* method used consists of the following four steps (Fig 1).

### Discover aspects of the community that impact healthy living

The CS were trained to use the mobile application tools. The first three schools used the Epicollect 5, and the other three schools used the Discovery tool to collect data. They were asked to take a “discovery walk” to i) identify barriers to PA and healthy eating, within their school, home and neighbourhood environments in the first set of three schools located in two middle/high SEAs and one low SEA and ii) identify barriers to PA, healthy eating, hygiene, and safety within their school environment only in the three additional schools located in low income areas. This was due to preliminary findings from the initial three schools, that schools located in low-income areas, are faced with more barriers in promoting and adopting healthy lifestyles and that safety and hygiene are critical factors for adolescents in low SEA schools when compared with their counterparts. The CS worked individually in the first three schools due to covid-19 pandemic, and in groups of twos and threes in the other three schools. The CS had the option of using their phones or the mobile phones provided by the research team to collect data. Telephone interviews were also conducted with the CS from the first three schools, who were recruited at the onset of the COVID-19 pandemic, and their caregivers (typically parents), to explore barriers to healthy diet and PA in their home, school, and neighbourhood environments. The interviews were conducted in any of three common languages in Cape Town (English, IsiXhosa, Afrikaans) based on participant’s preference and were translated and transcribed into English for analysis.

### Discuss and thematically analyse the findings

A qualitative framework analysis approach guided by an adapted setting- based socioecological framework (Individual, Home, School and Neighbourhood Domains) was utilised for the qualitative data analysis.

A preliminary analysis of the interview was conducted by the researchers. Two researchers familiarised themselves with the interview transcripts. An analytical framework was developed based on the study’s adapted socioecological domains (individual, home, school and neighbourhood) to organize the data. Findings were coded as subcategories within each domain category of the framework. Discrepancies in interpretation of codes were resolved through discussion and consensus among the coders and the research team.

**Fig 2:**
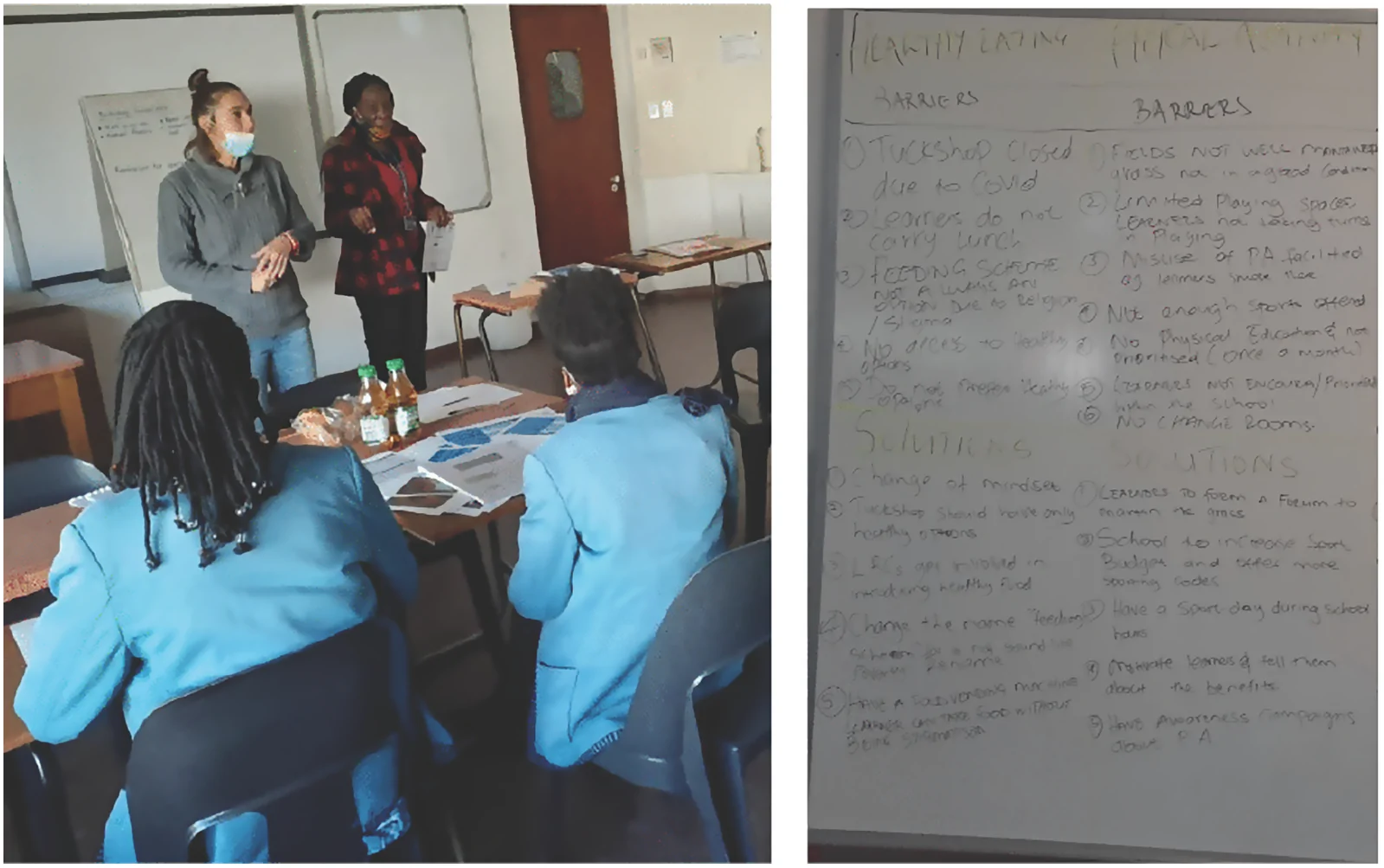
Discuss (The data validation and solutions workshop). This figure shows a citizen scientist validation and solutions workshop in session and how data was collected during the workshop.

In a researcher- facilitated citizen science validation and solutions workshop in each school, the CS were provided with unanalysed de-identified photographs they had taken with the mobile device and associated verbatim narratives and the list of subcategories within the adapted socioecological framework categories from the interviews. The groups of CS were asked to sort through these data in groups or individually and then in a plenary, the CS validated the data and contributed to refining existing categories and generating additional ones, all of which were categorized according to the study’s socioecological framework (Fig 2). Also, in plenary, the CS proffered perceived feasible solutions to the barriers categorised and suggested relevant changemakers that could champion the change. The final data categorisation and suggested solutions to the barriers were documented on a white board or flipchart during the workshop.

### Activate and advocate for local improvements

Champions from the CS group at each school were selected by their peers to present their findings and advocate for change to improve access to healthy and active living in their respective environments, especially the school environment. The CS champions worked with the research team to prepare their presentations and set up an advocacy workshop with the school staff in conjunction with the TLO to present their findings and proffer solutions. The CS were presented with certificates of participation as CS and were encouraged to continue to advocate for change for a healthy people and community. The CS and participating schools were encouraged to select a project for their school, based on their findings and the dialogue with educators and school leadership.

### Change stage

During the facilitated data validation and solutions and advocacy workshops, the CS and the TLO decided on a school-based intervention project that they felt was feasible and important to advocate for change. The CS, the school and the TLO worked together to identify at least one feasible proposed change from the data validation and solutions workshop to promote healthy and active lifestyles and environments within their schools. These changes were primarily led by the CS with approval from the school principal and support from the TLO and the research team. The intervention projects were organised by the CS with the assistance of the TLO, research team, and any necessary stakeholders. The CS and TLO were given a period of 6–12 months to plan the strategy for the school-based project solution, mobilise support to implement the strategy, monitor the progress of the project strategy, and as a team implement and execute the project. Each school could implement their own school-based project, and it included either one solution/change or many solutions/changes.

### Quantitative methods: Questionnaires and anthropometric measurements

Demographic characteristics of the CS such as gender, age, and years in residential location were collected using a questionnaire, as well as anthropometric measurements. Details of these quantitative measures have been published elsewhere [27].

### Analysis

Descriptive statistics for quantitative data were reported for the CS characteristics.

A qualitative framework analysis approach [36], guided by an adapted setting- based socioecological framework (Individual, Home, School and Neighbourhood Domains) was utilised for the qualitative data analysis. Analysis was conducted using NVivo qualitative analysis software version 12 and Microsoft Excel. Coding was done independently at each site by 2 researchers with cross-referencing of codes on a regular basis.

All data collected using the mobile applications were downloaded to two central servers once the CS completed their discovery walks. Researchers de-identified photographs and associated narratives of data collected with the mobile applications and printed them out for analysis in addition to the subcategories of barriers generated from the interviews for further in-depth. A detailed description of the qualitative analytic process has been described earlier in the “Discuss and analyse the findings”

The validated data in each school, which was collected through a uniform data validation and solutions workshop process and included only findings that were explicitly validated or agreed upon by the citizen scientists during these workshops were subsequently compared by two researchers and grouped according to similarities and differences across schools that collected the same types of data. Diet and physical activity data were collected across all schools, enabling a direct comparison between “low” and “middle/high” SEA categories. For hygiene and safety data, which were identified as unique challenges for low -SEA schools, the analysis was focused on the later three low SEA schools. Discrepancies of comparison were resolved through discussion and consensus among research team. Suggested solutions were not compared across schools, as each school focused on barriers perceived to be actionable within its own context steps rather than produce directly comparable findings across settings.

### Ethics approval

Ethical approvals were obtained from the University of Cape Town Human Research Ethics Committee: 088/2019; as well as approval from the Department of Education and the school principals of the selected schools. The study was conducted in accordance with the Declaration of Helsinki, applying the Society of Adolescent Health Guidelines for Adolescent Health Research pertaining to specific ethical considerations relevant to the protection of minors participating in research [37]. The written assent of each adolescent and consent from an adult parent or guardian (for those volunteers under the age of 18 years) was sought before data collection. The TLOs and Principals also signed the informed consent forms.

## Results

The results from this study are presented in several sections below. The first section presents the sociodemographic characteristics, the second section includes the four steps of the *Our Voice* method, i.e., Discover, Discuss, Advocate and Change which were categorised and compared based on the school’s SEA.

The sociodemographic characteristics and body mass index of the CS are categorised by their school SEA and are presented in Table 1.

**Table 1:** Sociodemographic characteristics and Body Mass Index (BMI) of adolescents in Cape Town categorised by their school socioeconomic area.

Variables	Low SEA School n = 36)	Middle/High SEA School (n = 29)	Total	P value
**Age [Median (Q1, Q3)]**				
Female (years)	15.0 (15.0,16.0)	15.0 (14.0,16.0)	14.7 (15.0,16.0)	0.66
Male (years)	14.5 (13.3,16.0)	15.0 (13.0;16.0)	13.0 (15,0,16.0)	
**Gender n (%)**				
Female	25 (37.9)	22 (33.3)	47 (71.2)	0.46
Male	12 (18.2)	7 (10.6)	19 (28.8)	
**BMI Categories n (%)**				
Underweight	1 (1.5)	0 (0)	1 (1.5)	0.63
Normal Weight	28 (43.1)	22 (33.8)	50 (76.9)	
Overweight / Obese	7 (10.8)	7 (10.8)	14(21.5)	
**Caregiver**				
Female	12(100)	20(83.3)	32(88.9)	<0.05
Male	0(0)	4(16.7)	4 (21.1)	

Abbreviation: Q1 = First quartile, Q3 = Third quartile, BMI = Body Mass Index; SEA: Socioeconomic area; P value=≤ 0.05.

A total of sixty-six (66) adolescent CS with median age of 15 years participated in the study, of which thirty-six (59.1%) were from low SEA schools and twenty-nine (43.9%) from middle/high SEA schools. In each of the schools, there were more participating girls (71.2%) than boys (28.8%) with no significant difference when categorised by school SEA. Approximately one in every five CS (21.5%) was overweight/obese regardless of the SEA schools.

### Discover

Table 2 provides information on the types and number of data that were collected during the discovery walk and the interviews.

**Table 2:** Discovery walk data and interviews conducted.

Variables	Low SEA School 1	Low SEA School 2	Low SEA School 3	Low SEA School 4	High SEA School 1	High SEA School 2	Total
Number of Photos	50	16	23	48	48	17	202
Number of Texts	47	11	19	32	45	17	171
Number of Voice Notes	6	0	1	11	7	0	25
Interviews Adolescents	13	0	0	0	15	10	38
Interview Caregivers	13	0	0	0	8	15	36

Abbreviation: SEA = Socioeconomic area.

**Fig 3:**
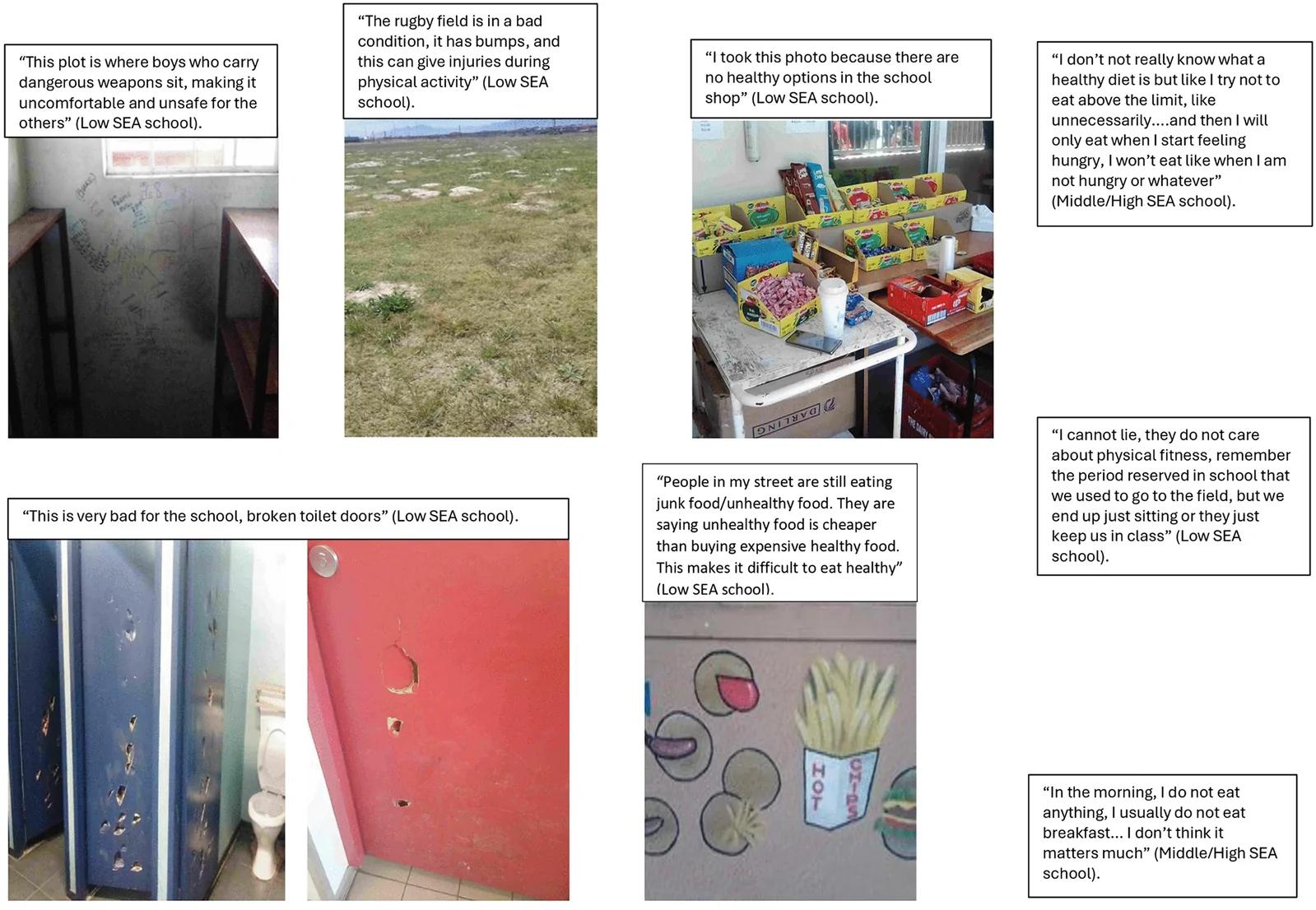
Photos and transcribed voice notes and text collected by adolescent citizen scientists. This figure shows samples of photos and narratives collected by the citizen scientists.

A total of 117 photos, 109 texts and 18 voice notes were collected by adolescents from the low SEA schools and 65 photos, 62 texts and 7 voice notes were collected from adolescents from the middle/high SEA schools. In addition, 26 interviews from adolescents from the low SEA schools and their caregivers and 48 interviews of adolescents from middle/high SEA schools and their caregivers were collected to better understand the barriers and facilitators of diet and PA. Examples of extracts from the data collected are shown in Fig 3.

### Discuss

Factors that are barriers to diet, PA, safety and hygiene in the school, home, and neighbourhood environments of urban adolescents in Cape Town, South Africa are presented in Tables 3–6. These also include the suggested solutions and changemakers. Changemakers were not identified in some of the suggested solutions.

**Table 3:** Barriers to healthy diet and proffered solutions identified by adolescents from low and middle/ high SEA schools.

Diet	Proffered Solutions	Changemakers
**School Domain**		
Healthy food options if available are expensive	• More affordable healthy food options such as fruits, sandwiches, yoghurt	School management
Limited or no healthy food options at Tuckshop	• More affordable healthy food options such as fruits, sandwiches, yoghurt • Available food vending machine for learners to conveniently select their meals at their leisure • Learners to participate in creating the tuckshop menu for healthy eatable and affordable options • Have the learner operate the tuck shop and making more healthy food options available • Adopt the Western Cape on Wellness government initiative tuck shop guideline of healthy food options in which the learners will decide on the types of foods to be sold at the tuckshop by a willing food contractor	School management, adolescents
There is limited information on healthy eating	• More health education in subjects such as Life orientation	School management
The tuckshop was closed due to Covid	• Change of mindsets regarding healthy food	Adolescent
Learners do not carry lunch from home.	• Access to healthy food options in the tuckshop	
^**#**^Feeding scheme food is not always an option for some due to religious reasons, or have allergies (no transparency during preparation, e.g., there might be pork).	• Learners to advocate and be involved in tuckshop healthy menus and negotiate for affordable prices. • A survey needs to be conducted for feeding scheme to accommodate allergies and preferences	School management
^**#**^Poverty stigma associated with eating the feeding scheme food	• Rename the feeding scheme to eradicate the stigma • Learners should be allocated a space to access the feeding scheme to limit learners looking or judging them • Have a food vending machine so learners can take food without being stigmatised	School management
Learners’ preference for unhealthy food options.	• Change of mindsets regarding healthy food	
Only one Tuckshop	• More food outlets to be provided in the school	
Mostly unhealthy food options sold at the school tuckshops	• Fruits, water and juice should be sold within the school	
No water is available at the Tuckshop. No food and water available for purchase, only snacks are being sold at school.	• More taps need to be installed for water availability. • Learners to advocate for fruit, salad and water availability in the school	School management, adolescent
Long lines at the tuckshop during break time	• More food outlets to be provided in the school • Separate break time for different breaks • Open all outlets/opening for the tuck-shop	
^**#**^Feeding scheme serves only sandwiches and there are no varieties/choices for those who might have allergies		School management
^#^Tuckshop is not available within the school as there is no facility built for it	• Learners with the aid of a representative council of leaders to advocate for the opening of a Tuckshop	Adolescent, school management
Breaks are too short (and not enough time to eat	• Longer breaks	School management
Lack of variety in tuck shop	• More varieties	
**Home and Individual Domain**		
Affordability of healthy food options. Healthy foods are expensive	• Home garden • Improvise • Buy food products at market day where there are cheaper	Adolescent and caregiver
Lack of autonomy of food choice. The learners have limited influence in food choice at home	• Educate parents	Adolescents
Personal choice of unhealthy food options (healthy foods are often not appealing)	• Find recipes that helps to make healthy food appealing or hidden, e.g., soup, smoothie • Cook in a way that makes healthy food appealing, Hide the veggies, find various food recipes	Adolescents and caregiver
Lack of time to eat breakfast	• Time management • School should start 9–4pm instead of 8am- to 3pm • Grab high fiber snacks such as nuts and raisins • Be more active and involved in shopping and shopping list for grocery	Adolescents; School management
**Neighbourhood Domain**		
Lots of unhealthy food options that are accessible and affordable	• Soup kitchen. • Community garden	Community committee
^**#**^Fresh foods are perishable	• Find ways of storing the fruits and vegetables appropriately	Adolescents and caregiver
Advertisement on social media and billboard	• Discipline	
^**#**^Limited access to healthy food options	• Be involved in shopping list	Adolescents
^**#**^Cost of healthy food options	• Buy food products at market day where there are cheaper	

#Unique barriers for adolescents from low SEA schools; ^*^Unique barriers for adolescents from middle/high SEA schools. Abbreviation: SEA = Socioeconomic area

**Table 4:** Barriers of physical activity and proffered solutions identified by adolescents from low and middle/ high SEA schools.

Physical Activity	Proffered Solutions	Changemakers
***School Domain***		
The sports fields are not maintained	• Learners can form a club that will maintain the field grass.	Adolescents, school management
^**#**^Limited playing spaces (learners not giving others a chance to play during break time). Limited to limited fields to play (e.g., there is always a rugby vs cricket battle/conflict due to sharing of field)	• The school should increase the sports budget. • Sports days for different sports must be separated with days or weeks for less conflict	School management
^**#**^Misuse of sports facilities (learners turned it into a smoking area and container classroom placed in sport space	• There should be a day dedicated to sports only	School management
Lack or limited of sports varieties	• Work with community coaches to assist and train learners • Have sports throughout the year • Have options of non-contact sports during the pandemic	School management
Physical Education (PE) is not prioritised in the school (frequency ranging from once a week to once a month)	• The school should Increase the allotted time for physical education classes and ensure they are fun	School management
^**#**^No changing rooms for sports within the school or the sports change rooms are not in a good state	• Change room clean-ups by learners • To create space in the change room, equipment needs to be moved to the storage	*School management*
No sport competition	• Privacy doors on toilets should be fixed • Physical Education should incorporate basketball • More sporting varieties to be introduced for variation and choices • Form relationships with other schools for competitive sport	School management
Limited PE and teachers and learners are not interested	• Physical education needs to be interesting by including activities such as dance etc • Learners should be educated about physical activities, its benefits and encouraged to participate in physical activity • Have varieties and creative of ways of during PE varieties by both the teachers and the learners • Teachers should be committed and make PE fun • Learners to be more committed, motivated and serious about PE	School management and adolescents
^**#**^No sport coaches		
^**#**^Uniform not appropriate for PA and sports clothes are not provided (Learners are allowed to bring their own PE clothes, however most of them do not have adequate clothes for PE except things like jeans etc)	• School to provide sport uniforms. They are willing to contribute to the funds as a sort of development fee for the uniform	School Management
Limited equipment and resources and varieties of sports for PA	• More variety of sports such as volleyball, dance • Have a market day to raise funds for sports equipment (fundraiser).	School management
More attention on some sports more than others	• Give equal attention to all sports	School management
**Individual and Home Domain**		
Limited space for exercise	• Improvise	
Time constraint for leisure exercise as time is occupied with house chores and schoolwork		
Laziness and lack of motivation	• Self-motivation • Create groups to motivate one another	
^**#**^No support or encouragement by family/ Lack of family support	• Create group to motivate one another • Educate the caregivers and other family members	Adolescents
Lack of motivation		
**Neighbourhood Domain**		
Unsafe environment and gender specific safety concerns	• Exercise in large groups	Adolescents
^**#**^No coaches		
^**#**^Dirt and pollution	• Write a report to the counsellor	Community committee
^**#**^Police harassment of group or people, usually male adolescent/youth	• Write a report to the counsellor	Adolescents, community committee

#Unique barriers of adolescents from low SEA schools

Abbreviation: SEA= Socioeconomic area; PE=Physical education; PA= Physical activity

**Table 5:** Barriers of hygiene and proffered solutions identified by adolescents from low SEA schools.

Hygiene	Proffered Solutions	Changemakers
***School Domain***		
^#^Lack of hygiene/sanitary stations and products in the school Limited sanitary bins in the restrooms, and those available are often unsanitary	• Learners to raise funds to purchase sanitary bins • The school should provide more sanitary stations • Learners need to make posters with instructions on how to dispose of sanitary towels appropriately • Learners and cleaning staff monitor refill of the hygiene products, e.g., soap, and toilet papers.	Adolescents, School management
Lack of adequate ventilation in the restrooms (no windows)		
^**#**^No soap or sanitisers in the toilets, Inadequate sanitizer dispensers in the toilets (Not enough sanitisers/soap)		
Lack of access to the restrooms during break time	• Learners are to be responsible for ensuring the restrooms are kept clean	Adolescents
^**#**^Lack of toilet paper in the restrooms	• Ask for more toilet paper during the interval.	Adolescents
^#^Unclean toilets (Toilets are not clean)	• Cleaning staff and learners need to keep the toilets clean • Learners to form a clean-up initiative to clean paper/plastics and toilets in the school. • Hygiene awareness campaigns.	School management, adolescents
No sanitary pads available at school.	• Initiative/campaign to raise funds and request for donors from businesses/teachers/parents for identified project	Adolescents
^**#**^Inadequate trash bins and those available are unclean	• Clean trash bins need to be placed in every corner of the school • Increase awareness about the importance of recycling to minimize trash.	School management, adolescents
^**#**^Water taps and toilets are broken; Broken water fountain and the water look unclean, Water was only accessible in the bathrooms	• Learners to make sure the school committee/management is aware about these issues and seek external help • Make the school management aware of the broken fountain water and hygienic concerns.	Adolescents
Learners smoke cigarettes/dagga in public spaces, and the smell stays on the non-smokers uniform as well.	• Disciplinary committee to be formed to punish smokers, and the consequences to be consistent.	School management

^*#*^ *Crosscutting barriers of adolescents from low SEA schools*

**Table 6:** Barriers of safety and proffered solutions identified by adolescents from low SEA schools.

Safety	Proffered Solutions	Changemakers
***School Domain***		
^**#**^Unsafe environment outside the school gate, learners are at risk of being robbed	• Advocate for the learners to wait for transport inside the school • More security personnel at the gate or police patrol	School management
Gangsterism is a challenge within school; The school's security measures are inadequate, allowing gangs to gain access Learners easily carry dangerous weapons with them	• Reinforce the school’s security to prevent gangsters from accessing the school premises • The management should discipline troublemakers adequately • The school should re-introduce scanning for weapons • Thorough screening of learners for weapons	School management
Lack of CCTV cameras in the school	• Learners can organise anti-violence campaigns within the school	Adolescents
Racism conflicts among learners, yet the school fails to adequately address and discipline those responsible	• Teachers to monitor the learners during intervals	School management
#The school infrastructure is old and requires repairs, exposed electric wires, Drainages are not well installed; learners can fall inside when stepping on them, Hazard signage should be placed near dangerous drainages to prevent harm to learners The school needs to fix the damaged drainages	• The school to replace the old infrastructure, parents with construction skills can assist with repairs. • Hazard signage should be placed near dangerous drainages to prevent harm to learners • The school needs to fix the damaged drainages • Learners to maintain the playing ground by filling the holes with sand	School management
	• Maintenance committee/staff required	School management
Theft among learners	• There needs to be a policy in place to punish theft.	Adolescents, School management

^*#*^ *Cross-cutting barriers of adolescents from low SEA schools*

### Healthy diet barriers and proffered solutions and strategies

Table 3 shows the barriers and proffered solutions to healthy diets identified by adolescents from the low and middle/high SEA schools. The barriers were further compared by the school SEA and classified as either crosscutting across the schools regardless of SEA or unique (one school or more) to SEA specific schools. There were several common themes of barriers to healthy eating across the schools regardless of their socioeconomic status. Within the school environments, these included limited health education, the lack of availability of healthy food options in the school tuck shops and vendors, and where such food is available, it is unaffordable, lack of food variety in some cases, and no tuck shop or food service during the Covid-19 period. The adolescents also cited long queues to access food, which limited available time during breaks. A preference for unhealthy food, unappealing appearance at healthy foods, laziness to prepare healthy lunch from home and the influence of unhealthy food choices due to peer pressure were also cross-cutting barriers of healthy eating. A phenomenon that was reported, unique to schools from low-income areas, was the perception that the school feeding nutrition support programme is stigmatising and not acceptably prepared to suit religious food requirements (e.g., not Halaal), and the tuckshop was situation outside the school environment Adolescents from both low and middle/high income areas identified barriers to healthy eating in the community/neighbourhood such as unhealthy food options being more accessible and cheaper, and within the home environment, citing lack of autonomy over their food choice decisions. Unique to the adolescents from low-income areas were the limited access to healthy food choices in their environment and unaffordability of healthy food options.

The adolescents identified targeted, and feasible interventions to the barriers of healthy eating. Several themes emerged as possible strategies to address the lack of availability of healthy foods in tuck shops. These included learners creating a healthy menu or even participating in operating the tuck shop. Healthy and affordable food options such as sandwiches, yoghurt and fruits that could be sold were identified. Other suggestions were aimed at reducing the stigma associated with the school feeding scheme, including renaming the feeding scheme programme, providing privacy to those who wish to access the service and providing improved health education. In most of these strategies, learners identified the school management as the change makers, including strengthening the health education curriculum. However, they identified themselves as the change makers in helping to alter the mindsets of their peers towards healthy foods, although no specific strategies were offered to address this barrier. In terms of accessing affordable healthy food at home or in the community, some learners suggested food gardening and soup kitchens as a means of lowering barriers to healthy food. Having the adolescents more involved in the shopping list and educating their parents/caregivers were some suggested solutions to the lack of autonomy of food choice in the home. The adolescents in both low and middle/high SEA also indicated that they did not mind eating healthy food if they are hidden in the meal (e.g., made into a smoothie, blended with other ingredients). In one of the schools, they came up with the slogan “hide the veggies” (Table 3). Changemakers were not identified in some of the proffered solutions.

### Physical activity barriers and proffered solutions and strategies

Barriers to PA and solutions identified by adolescents from low and middle/high SEA schools are presented in Table 4. The barriers were further compared by the school SEA and classified as either crosscutting across schools regardless of SEA or unique (one school or more) to SEA -specific schools. Adolescent CS across all the schools expressed concern over the low priority attached to PA and physical education in the schools. In some cases, Physical Education periods were limited to once a week to once a month sometimes. The adolescents, regardless of their SEA schools, identified that there lack of equipment, poor condition of sports fields and lack of space (or appropriation of space for other purposes), limited number of sports choices at their school, little or no opportunities to participate in sports competition. Prioritization of some school sports compared to others were also identified as cross-cutting barriers, although there were more varieties of sports and sports fields in the middle/high income schools in comparison to their counterparts. Unique to individual low-income schools were dissatisfaction that the school uniforms were unsuitable for sports participation, lack of sports coach and sport change room inadequacies misuse of sport designated facilities such as placement of a school classroom in a sports zone and using sport court or field as a sit out and smoking area.

Safety was cited as a cross-cutting limitation to participation in PA, but emphasized more within the low-income areas. During the thematic analysis in one of the low-income schools, the adolescents shared their experience of groups of young men walking or exercising in the community being harassed by police who misinterpreted their intent. They also cited the lack of community-based coaches. Within the home environment, unique to adolescents from low-income SEA was lack of support from their family to participate in desired PA, especially those sports activities that are culturally frowned upon for ladies such as yoga, and dirt and pollution in the neighbourhood. There were no unique PA barriers for the middle/high SEA schools.

As potential solutions to some of these barriers for improving PA, there was strong support for inter- and intra- school competitions, and CS felt that there would be greater uptake of physical education and PA with a broader range of activities including dance and soccer. The CS indicated that physical education should receive greater priority by school management and involve learners in co-creating engaging activities. There were also suggestions that the teachers should also show interest and motivation as often their physical education session was used to sit, talk or just hang out with friends. There was support for developing relationships with other schools to increase opportunities. These solutions were largely relegated to the school management as “change makers”. However, more than one citizen science group identified that the learners should be willing to engage with sports field clean up and maintenance. It was also suggested that more emphasis should be placed on PA as part of the health education curriculum, and that learners also needed a mindset shift toward PA and physical education. The “change makers” identified were the adolescents, school management and the physical education teachers. With respect to PA in the community, in terms of pollution and rubbish, safety and harassment, CS suggested writing letters and advocating to their city councillors.

### Hygiene barriers and proffered solutions and strategies

Table 5 presents the hygiene barriers and proffered solutions identified in the low SEAs schools. The barriers were further compared within the low SEA schools and classified as either crosscutting across the Low SEA schools or unique (one school) within the low SEA schools. Lack of, or insufficient functional, clean toilets, toilet paper, sanitary bins, access to fresh potable water in the school were raised as challenges in all the low-SEA schools. Most of the responsibility for these concerns was placed on the school management, but there was recognition of the learners’ responsibility to maintain the facilities, if they were improved or upgraded. In one of the schools, it was suggested that learners could become involved in clean up campaigns to support school management efforts or fund-raising efforts for better sanitation. Other suggestions were learners making posters to raise awareness regarding the co-responsibility to maintaining school hygiene. The CS highlighted the need for communication to school authorities regarding quality of and availability of potable water. Unique hygiene barriers specific to a low SEA school include, lack of access to restroom, inadequate ventilation in the restroom and smoking on campus and the need for more consistent discipline in terms of smoking. Dirt and pollution were the primary hygiene issues highlighted in the neighbourhood. (Table 5).

### Safety barriers and proffered solutions and strategies

Table 6 presents the safety barriers and proffered solutions identified in the low SEAs schools. The barriers were further compared within the low SEA schools and classified as either crosscutting across the Low SEA schools or unique (one school) within the low SEA schools. Most of the barriers were unique to a low SEA, except for the poor maintenance and repairs of infrastructure in the all the schools and there was general concern in low SEA schools over the unsafe environment out the school. The unique barriers ranged from gangsterism, racism and theft in the school with suggestion solutions such as harsher punishment for learners who impact the security and well-being of security services be employed on school premises and scanning for weapons at school entrances should be introduced. There was also a sense that the gangsterism and anti-social problems of the surrounding neighbourhood affected the school, and that increased security and a parents/learners/school management meeting may help to find solutions. In one of the schools, an anti-violence campaign within the school was suggested as an intervention (Table 6).

### Advocate and Change

As a critical next step following the discuss step which involved data analysis, validation and actionable solutions brainstorming, all the six schools chose advocate champions (representatives of the CS in each school) who worked with the liaison officer and the research team to showcase the citizen science project. These were findings from the thematic analysis and their proffered solutions of the school environment to the school staff or representatives of the school staff in an advocacy workshop. Importantly, there was allotted time for the school staff or representatives to provide feedback and to identify key actionable steps. During the advocacy workshop, the CS were also awarded with a certificate of participation as CS and a gift for their time and participation. Proposed action steps for each of the schools were to identify at least one feasible change that could be championed by the CS and the schools.

**Fig 4:**
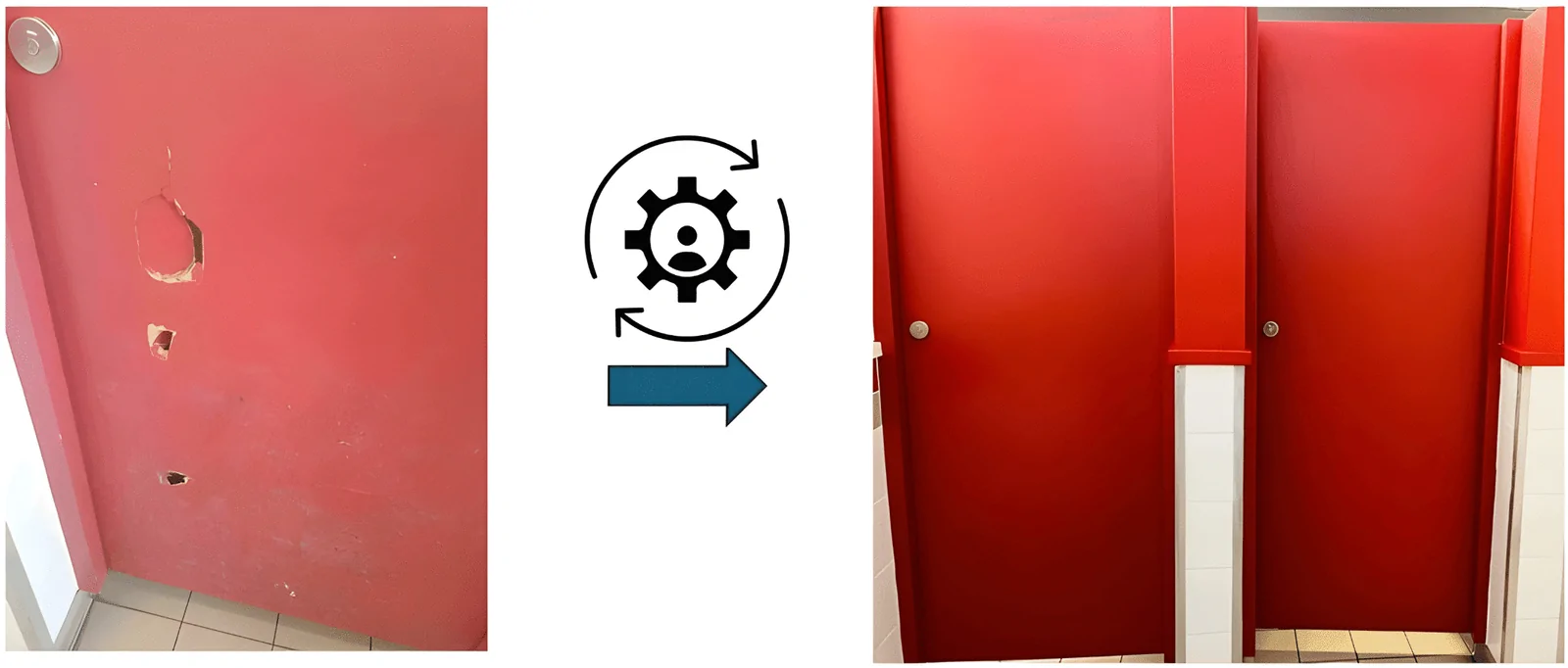
Infrastructure repairs. This figure shows a change resulting in the repairs of damaged toilet doors.

Of the six schools, five (One from middle/high income areas and four from low-income areas) completed projects or activities arising from their engagement in the *Our Voice* method. One of the schools organised and implemented an intra-school netball and soccer competition, complete with prizes, galvanising and energising the entire school. Another school raised funds and awareness around feminine hygiene products. One school selected a recycling project, focusing on environmental health and awareness, and the fourth school held games and PA events to create awareness and fund raising for sports equipment. Other changes led by the school management as a result of the citizen science project included the sinking of a bore hole for the school as a solution to poor portable water, cultivation of a school garden to promote PA and access to healthy and affordable fruits and vegetables, repairs of toilet doors (Fig 4), and improved hygiene and replacement of trash cans in the school environments in three of the low income schools. These and other ripple effects of the citizen science project will be reported elsewhere.

## Discussion

Our study which is grounded in participatory action research, socioecological and health promoting frameworks highlights various elements. It shows the barriers to healthy and active lifestyles and environments (diet, PA safety and hygiene) that impact South Africa adolescents from low and middle/high SEA schools in Cape Town across setting-based socioecological domains. It underscores the importance of engaging those who are most impacted by the challenges (in our study, adolescents) as part of the solutions/ interventions. Furthermore, the study emphasizes the critical role that adolescents and changes in the school environment can play in improving active and healthy lifestyles and environments for adolescents in their immediate environments, especially the school environment. It exemplifies how schools can be aligned to the HPS framework by strengthening health promotion within school settings and enabling and empowering adolescents to be active agents of change. It also showcased how adolescents can be guided to collect meaningful and high-quality data, analyse those data, and successfully advocate for and be empowered to make positive change within their immediate environments. Our study also highlighted that while there were many common barriers of diet and PA to health across populations, low-income communities face additional, unique obstacles linked to economic and structural resource limitations peculiar to their context.

In our study, the opportunity for the adolescent CS to share their self- identified barriers to healthy and active environments related to diet, PA, safety, and hygiene provided the opportunity for the school as a safe and enabling space as core component of the HPS framework to hear the voices of those most impacted by those barriers. For these adolescents, it was the first time they had the opportunity to have a voice and be part of a change process such as the *Our Voice* citizen science method. In our study, adolescents were immersed and were part of the research process. The *Our Voice* method brought about strong participation and commitment and created a sense of ownership and achievement. This study also provided the opportunity for the adolescents to work collaboratively through collective action with support from the research team and the school staff.

Many of the common barriers to PA such as lack of competitive sport, limited personal interest/motivation, lack of prioritization of PE in schools along with only modest interest/engagement of PE teachers, and inadequate sports varieties and facilities, are comparable to results from several studies in LMICs [38]. Appropriately designed PE has been identified as critical to improving PA as a lifestyle [39]. Furthermore, adolescents with adequate physical education, motivation, and competition are more likely to engage in increased PA [39]. For many adolescents, PE in the schools is oftentimes the only opportunity to learn and engage in PA, and PE teachers can play a major role in motivating and engaging the learners [39–41].

The school environment is a relatively safe environment to promote healthy lifestyles and activities, especially PA. Various studies have indicated the lack of prioritization of physical education in high schools in African countries [42,43]. Our study emphasized that many of the CS perceived the PE period in school as a free time to sit and chat with friends, as some of the PE teachers were not interested or motivated to educate or engage them in PA and that the activities available were not engaging.

The low- SEA home and the neighbourhood were perceived as being limiting to PA in terms of limited space and lack of support from family, which have been similarly noted by other studies in LMICs [32,44,45]. Interestingly, safety in the environment was a barrier to PA regardless of the SEA. While many studies have emphasized more safety challenges in low SEA as a critical barrier to PA [46,47], especially for females, South Africa has a high crime rate overall which may be a contributory factor to this cross-cutting barrier [48,49]. A unique type of safety barrier identified in one of the low SEA schools was male-specific safety challenges, i.e., police harassment of groups or people, usually male adolescent/youth when exercising. Unfortunately, the law enforcement that is meant to be an emblem of safety and protection in the communities is seen as a threat instead [50]. Also, similar to another study [26], dirt and pollution/poor aesthetics are important barriers to PA in the low-income areas in Africa. Other unique barriers in low SEA schools such as sociocultural influences (e.g., some girls were not allowed by their parents to do certain PA activities such as yoga) have been highlighted in other LMICs studies [47,50].

Commonly identified barriers of adolescents’ healthy eating such as limited health education, lack of motivation, preference for unhealthy food, limited time to eat breakfast, not packing lunch to school, peer influence promoting unhealthy eating, easy access to unhealthy access in neighbourhoods, lack of autonomy, and the unappealing nature of some healthy foods are similar to findings from previous studies in South Africa [51,52] and in other LMIC countries [53]. Life orientation is a mandatory subject in South African high schools and aligns with the HSP framework by providing structured health education within the classroom, promoting learners’ physical, mental, and social well-being [54,55]. However, studies have supported our findings of limited health and physical education, as LO is often considered irrelevant by learners, alongside systemic barriers such as inadequate teacher training, resource constraints and misalignment of the curriculum with its set objectives ([54,55].

Unique barriers identified in low-income schools such as stigma and lack of consideration for cultural/religious beliefs and dietary restrictions of the free school feeding scheme, i.e., the National School Nutrition Program (NSNP) found in our study are supported by other studies conducted in South Africa [56–58], suggesting that these challenges remain a problem. The NSNP provides school meals to primary and secondary public schools in South Africa, often in the low-income categories. Notably, South Africa provides NSNP based on quintile categorisation of Quintile 1(Q1) to Quintile 5(Q5). Q1 denotes the most disadvantaged schools, progressing to Q5 as the least disadvantaged schools. Oftentimes, only Q1 to Q3 benefit from NSNP, which may be a contributory factor to why this was not seen as a barrier for the middle/high SES schools (often categorised as Q4 and Q5), as they are oftentimes not a beneficiary of the NSNP [56–58]. This circumstance might itself be a contributory poverty stigma for those in the low-SEA schools. Other unique barriers for adolescents from low SEA schools are the lack of access to healthy food options and affordability issues in their home and neighbourhoods, which are not unexpected and match other studies conducted in South Africa and other LMICs [53,59].

Lack of portable water, clean toiletry sanitary resources such as trash bins, access to sanitary items for girls, availability of hand sanitizers, and the occurrence of adolescent smoking in the schools, were the core barriers to hygiene in the schools, which are also supported by studies in other LMICs [60,61].

Often in Africa, the adolescent period is an under-optimised stage of untapped resources and potential for advocacy and interventions for healthy lifestyles and behavioural improvements. This study is the first *Our Voice* project that was completed with adolescents in Africa in which they participated in all the stages of the OV method, the outcomes of which show the profound impact that adolescents can make in advancing positive change within their environments, especially within the school community. One notable example of this was the sport equipment fund-raising activity developed through creative means using available resources.

Healthy and active lifestyles and environments are critical to reducing the prevalence of non- communicable diseases and their risk factors such as obesity, unhealthy diet and physical inactivity among adolescents and ensuring their well-being into adulthood. It is critical to provide platforms for adolescents to be involved in being part of the change for health, especially those that impact them. Evidence supported by the HSP framework has indicated that if adolescents are empowered to advocate for their health and that of their loved ones, they may become part of critical sustainable changes and interventions that may translate into emerging and future policy and practice and the creation of sustainable health-promoting school environments over time [62]. Importantly, this study and its findings align with the World Health Organization Health Promoting Schools framework and participatory action research by engaging adolescents as active change agents in the co-creation of school-based health initiatives that cut across socioecological domains with proximal and potential sustainable positive impacts.

### Recommendations

It is recommended based on our findings and supported by HPS framework, that schools as a critical environment in the development and change process of adolescents, should be a key component of promoting healthy lifestyles and environments in adolescents. The results support and recommend initiatives in which adolescents are empowered to act as change agents within their school environment and broader social environments. By equipping adolescents with skills, confidence, and opportunities for leadership, these initiatives can foster meaningful change beyond the classroom, extending to families, peer groups, and communities. Furthermore, it will promote equity, inclusion, and sustainable health promotion practices. To more fully understand the impacts of such adolescent-engaged citizen science initiatives, it is recommended to evaluate the longer-term “ripple effects” that such programs can foster, including how adolescent-led change influences norms and practices in their immediate environments.

Fostering a more comprehensive life orientation curriculum in South African high schools that is embedded in the HPS framework for the holistic development of learners that encompasses subjects like physical education, life skills, health education, and career guidance can be a more positive experience for all and has the potential for inspiring lifelong learnings through motivating and engaging learners in activities that are co-created with learners themselves.

### Strengths and limitations

This study is the first *Our Voice* project that was completed with adolescents in Africa in which they participated in all the four stages of the OV method. Also, the thematic analysis, advocacy and interventions were conducted with the adolescents to ensure a holistic perspective embedded in socioecological and HPS frameworks. Possible limitations identified are i) the heterogeneity in data collection across the schools. Interviews were conducted in only three of the schools with adolescents and their caregivers to support the data collected via the mobile application during the covid-pandemic. Also, although two mobile applications were used in data collection, both captured factors using comparable instructions and prompts. Additional variables (i.e., hygiene and safety) were introduced in three schools following preliminary data analysis from the first three schools, resulting in partial data availability for those indicators. However, all data were ultimately interpreted through data validation and solutions workshops in which the CS collectively analysed and validated the results. ii) As the study was conducted in specific contexts, the findings may not be generalisable to other settings.

## Conclusion

Our study’s findings suggest that the association of lower socio-economic status both at the individual and community levels, with additional structural and resource related barriers may be significant contributor to barriers to physical activity, healthy eating, safety, and hygiene. However, a surprising number of similar barriers were experienced by adolescents regardless of their school SEA. The buy-in and participation of adolescents and the school authority in these types of “research-to-action” activities to promote healthy lifestyles and environments is imperative. The study emphasizes the critical role that adolescents and changes in the school environment can play in improving active and healthy lifestyles and environments for adolescents in their immediate environments, especially the school environment. We conclude that the *Our Voice* citizen science method is a valuable multi-layered tool for empowering adolescents as advocates in promoting healthy and active lifestyles and environments in fostering impactful, health-enhancing contextual changes.
