# Supplementary material for: Adolescent-led Our Voice method to promote healthy and active lifestyles and environments in South Africa
Source: PLOS Glob Public Health. 2026 Sep 2;6(9):e0007141. doi: 10.1371/journal.pgph.0007141 (PMC13537583; doi:10.1371/journal.pgph.0007141)
Supplement: Supplementary file 1 [file pgph.0007141.s001.docx]

**Journal:** PLOS Global Public Health
**Manuscript ID:** PGPH-D-25-03964
**Title:** Adolescent-led Our Voice method to promote healthy and active lifestyles and environments in South Africa

**Essential Revisions**

**1. Heterogeneity in Data Collection Across Schools**

**Location:** Methods (Lines 153–161) and Limitations (Lines 528–530)

The scope of data collection differed across schools:

- Only 3 schools collected home/neighbourhood data.
- Hygiene and safety data were limited to low-SEA schools.
- Two different mobile tools were used.

While partially acknowledged in Limitations, the implications for cross-SEA comparison are not explicitly stated.

Better to insert a clarifying sentence in the Methods for logic behind this difference in methodology (Section 2.3.2 or 2.4):

**2. Qualitative Analysis Transparency**

**Location:** Lines 213–218

The description of thematic analysis is brief. It is unclear whether:

- An inductive approach was used,
- Themes generated by citizen scientists were triangulated with researcher coding.

**Expand analysis description by 2–3 sentences stating type of qualitative analysis, theme generation and coding procedure.**

**3. Cross-SEA Interpretation**

**Location:** Lines 534–537

The statement that lower socioeconomic status “is a significant contributor” may overstate findings from primarily qualitative data where “significance” in statistical terms cannot be measured.

Instead of **“Lower socioeconomic stat… is a significant contributor…” write “findings suggest association of lower socio economic status with additional structural and resource related barriers”**
