# Supplementary material for: Adolescent-led Our Voice method to promote healthy and active lifestyles and environments in South Africa
Source: PLOS Glob Public Health. 2026 Sep 2;6(9):e0007141. doi: 10.1371/journal.pgph.0007141 (PMC13537583; doi:10.1371/journal.pgph.0007141)
Supplement: Supplementary file 2 [file pgph.0007141.s003.docx]

**Reviewer Report (Round 2)**

**Manuscript ID:** PGPH-D-25-03964R1
**Title:** Adolescent-led Our Voice method to promote healthy and active lifestyles and environments in South Africa
**Journal:** PLOS Global Public Health

**General Assessment**

The authors have made **substantial efforts to address prior reviewer comments**, particularly in clarifying methodology, expanding the discussion, and improving transparency regarding data collection heterogeneity. Most of the **claimed revisions (pp. 91–100)** are indeed implemented in the manuscript.

However, the revision remains **predominantly additive rather than analytical**. The manuscript continues to fall short in terms of **qualitative methodological rigor and depth of analysis**.

**Major Comments**

**1. Insufficient Qualitative Rigor and Transparency**

**Issue**

The manuscript describes thematic analysis but lacks key methodological components expected in qualitative research.

**Specific Comments**

- **Lines 226–230 (Analysis section):**
  - Coding is described as being conducted by two researchers, but:
    - No description of **coding framework/codebook structure**
    - No mention of **inter-coder agreement or reconciliation process**
    - No indication of **data saturation**
- Provide:
  - A brief **coding framework or thematic structure**
  - Description of how disagreements were resolved
  - Statement on whether **thematic saturation** was achieved
- **Lines 188–193 (Thematic workshop):**
  - Participant validation is mentioned but lacks detail.
- Clarify:
  - How participant validation influenced final themes
  - Whether any themes were modified or rejected based on participant input

**2. Heterogeneity in Data Collection Not Analytically Addressed**

**Issue**

Different schools contributed **different types of data** (e.g., interviews vs no interviews; additional domains introduced later).

**Evidence**

- **Lines 149–151, 553–555**
- Interviews conducted only in initial schools
- Hygiene and safety introduced later
- **Major Action Required:**
- Either:
  - Stratify findings by data availability (early vs later schools), OR
  - Explicitly justify why pooled analysis remains valid

**3. Limited Analytical Depth of Results**

**Issue**

Results (Tables 3–6; Lines 289–374) are largely **descriptive listings** of barriers and solutions.

**Concerns**

- No hierarchy or prioritization of themes
- No cross-domain synthesis
- Limited interpretation of **why differences exist between SEAs**
- Add:
  - A short **analytical synthesis paragraph per domain**
  - Explicit comparison between **low vs middle/high SEA**
  - Identification of **dominant or recurring themes**

**4. Socioecological Framework Not Operationalized**

**Issue**

Although the socioecological model is referenced (Lines 72–74, 226–227), it is **not used analytically**.

- Explicitly map findings across levels:
  - Individual
  - Interpersonal
  - Institutional (school)
  - Community
- Consider adding a **summary figure or table**

**5. Overextension of Conclusions**

**Issue**

The manuscript claims effectiveness of the Our Voice method.

**Evidence**

- **Lines 44–46 (Abstract)**
- **Lines 430–443 (Discussion)**

**Concern**

- Study is exploratory/pilot without comparative evaluation
- **Major Action Required:**
- Moderate claims:
  - Replace “effective” with “feasible” or “promising”
  - Clarify absence of causal inference

**6. Methodological Justification for Tool Change**

- **Lines 154–160**
  - Tool switch is explained
- Add 1–2 lines on:
  - Potential impact on **data comparability**

**7. Quantitative Component Underdeveloped**

- **Table 1 (Lines 256–264)**
- Clarify:
  - Purpose of quantitative data in mixed-methods design
  - Whether it informs qualitative findings

**8. Integration of Advocacy/Change Outcomes**

- **Lines 415–425**

**Issue:**

- Intervention outcomes are described narratively but not analyzed
- Add brief interpretation:
  - What enabled successful implementation?
  - Differences across schools?

**9. Language and Clarity**

- Minor grammatical inconsistencies throughout
- Example:
  - **Line 28:** “Fostering adolescents’ agency and empowerment, can drive…” → remove comma

**10. Redundancy in Discussion**

- **Lines 455–470**
  - Repetition of known LMIC barriers

**11. Table Presentation**

- Tables 3–6:
  - Very dense
- Highlight **key themes** or summarizing in-text

**Verification of Author Claims (pp. 91–100)**

| **Claimed Revision** | **Status** | **Comment** |
| --- | --- | --- |
| Expanded theoretical framing | ✔ Done | Still descriptive |
| Clarified methodology | ✔ Done | Needs more rigor |
| Addressed heterogeneity | ✔ Partially | Not analytically resolved |
| Strengthened results | ✔ Partially | Still descriptive |
| Expanded discussion | ✔ Done | Needs critical depth |
| Updated data availability | ✔ Done | Adequate |
