# Supplementary material for: Adolescent-led Our Voice method to promote healthy and active lifestyles and environments in South Africa
Source: PLOS Glob Public Health. 2026 Sep 2;6(9):e0007141. doi: 10.1371/journal.pgph.0007141 (PMC13537583; doi:10.1371/journal.pgph.0007141)
Supplement: Supplementary file 3 [file pgph.0007141.s004.docx]

April 14, 2026

Dear Editor,

Many thanks for the constructive comments you have sent us. We sincerely thank the reviewers for their careful reading of our manuscript and for providing constructive feedback and suggestions. We have considered each suggestion and have compiled our responses to all the comments.

Full details of our responses to the comments are below and tracked changes in the manuscript. The line numbers included in the table below align with the manuscript revision in tracked changes.

With best wishes,

Dr. Feyi Wayas and Co-authors

| **Reviewer 1 Comments** | **Response to reviewer** |
| --- | --- |
| The manuscript addresses a highly relevant and timely topic in global public health by examining an adolescent-led, school-based intervention aimed at promoting healthy and active lifestyles in a context that is often underrepresented in the literature.  The study is innovative in both its methodological approach and its ethical stance, particularly by amplifying adolescents’ voices in settings where young people’s perspectives are frequently marginalized. The decision to move beyond the identification of barriers and to explicitly advocate for change further strengthens the public health relevance of the work.  Despite these strong points, several recommendations and suggestions should be addressed to further strengthen the rigour, coherence, and conceptual positioning of the manuscript before it can be considered for publication in PLOS Global Public Health. | Thank you for your comment and for the suggestions to improve the manuscript. |
| The introduction section of the manuscript is brief and have room for improvement. It would benefit from more explicitly foregrounding Participatory Action Research (PAR) as a central theoretical and methodological pillar of the study. Although PAR is mentioned, its role is not sufficiently highlighted given that the Our Voice method is a well-established and consolidated participatory approach rather than merely a data collection tool. Expanding the introduction to better articulate the contribution of PAR to school-based health promotion would strengthen the conceptual framing of the study.  Relatedly, it is recommended that the authors situate their work more clearly within the existing literature on PAR by incorporating recent systematic reviews or meta-analyses that examine participatory and action-oriented methodologies in youth or school-based health promotion. In the same vein, given the strong use of photo-based evidence and environmental documentation, it would be appropriate to reference key studies on photo-elicitation methods (e.g., Photovoice), which align closely with the role attributed to adolescents as citizen scientists in this manuscript.  The manuscript also implicitly aligns with a salutogenic approach to health and with the principles of Health-Promoting Schools. Given the strong emphasis on agency, empowerment, and sustainability, the authors are encouraged to position their work more explicitly within this framework. Referencing key international bodies such as the World Health Organization or UNESCO would provide a stronger external theoretical anchor and reinforce the global relevance of the study. The advocate for change component, in particular, would benefit from being explicitly linked to salutogenic principles and adolescent agency.  Another distinctive strength of the study is its multi-component intervention design. This feature could be more clearly articulated in the introduction and supported with references to prior literature on multi-component school-based health promotion interventions. Doing so would help contextualise the intervention and clarify how the present study extends existing approaches. | Thank you for your suggestions in strengthening the Introduction of the study. We have incorporated information about Participatory Action Research as a methodological pillar of the study, the Our Voice method which is further described in the methods. We have also incorporated details about the alignment of the study with the school-based health promotion framework and multi-component school-based health promotion interventions. The revised information is in the Introduction section (Line 51-113). |
| Methods: From my view, the manuscript may not need to be framed as a mixed-methods study. While the descriptive quantitative data are appropriate, the primary contribution of the article clearly lies in the qualitative analysis and in the participatory processes involving adolescent citizen scientists. The quantitative elements appear largely descriptive and do not substantially inform the interpretation of the findings. Presenting the study explicitly as a qualitative PAR-based investigation may therefore provide greater methodological coherence. Notably, the “Discover” phase, where evidence is quantified, contributes more to qualitative pattern identification than to inferential quantitative analysis. | Thank you for your comments. We have reframed the study as a qualitative participatory action research-based approach, “Our Voice: method supplemented with descriptive quantitative analysis. The section now reads as:  “The study employed a qualitative participatory action research-based approach, the Our Voice method (15, 22), supplemented with descriptive quantitative analysis” (Line 141-142). |
| Results: Regarding the results, the credibility and transferability of the findings would be substantially strengthened by including verbatim excerpts from the adolescent participants. Integrating direct quotations, either within the results narrative or as an additional column in the tables, would provide clear evidence that the reported themes genuinely emerged from the adolescents’ lived experiences, rather than being solely the result of researcher interpretation. This addition would significantly enhance the trustworthiness of the qualitative analysis and is considered one of the most important areas for improvement. | Thank you for the comment  Verbatim excerpts are included in Fig 3, which presents data samples collected. Fig 3 contains photographs the citizen scientists took along with verbatim descriptive excerpts.  In Tables 3–6, we presented the results of the thematic data analysis workshops. Researchers facilitated the discussions; however, the outcomes shown in the Tables reflect the workshop conversations exactly as they occurred, rather than the researcher’s interpretation. The themes were captured directly from adolescents during the workshops.  To clarify and to enhance the trustworthiness of the analysis, we have   - included a sample photo of an actual flipchart where the discussion of the thematic analysis workshop was captured (Fig 2). - We have included more information in the methods to highlight that the results shown in this study emerged from the adolescent’s lived experience in Line 187-194 which reads as:   “In a facilitated thematic analysis workshop, which was convened and facilitated by the research team, the CS were provided with de-identified photographs they had taken with the mobile devices and associated verbatim narratives and themes from the interviews. The groups of CS were asked to sort through the various photographs, verbatim narratives and themes generated through researcher coding of the interviews. The CS validated themes and contributed to refining existing themes and generating additional ones (Fig 2). Once these themes had been identified, the groups were asked to prioritize those factors which they felt were barriers or facilitators and, more specifically, proffer feasible solutions to the barriers and suggest which types of changemakers could champion the change” |
| Discussion: The manuscript would benefit from moving beyond a predominantly socio-ecological interpretation toward a more explicit salutogenic framing. The strong emphasis on advocacy, sustainability, and student agency offers an excellent opportunity to articulate how adolescent-led participatory interventions contribute to the creation of health-promoting school environments over time. Making this conceptual shift more explicit would strengthen the theoretical contribution of the study. | Thank you for your insights. We agree that the study aligns with the HPS framework, in addition to the socioecological framework and PAR approach, although the core framework is grounded in the socioecological framework and PAR approach. Therefore, we have provided more informative interpretation in alignment with the HPS framework with emphasis on advocacy sustainability and student agency in the discussion section from Line 430-524. |
| Finally, in the recommendations section, greater emphasis could be placed on adolescent empowerment and the development of agency. Highlighting how this approach contributes to equity, inclusion, and sustainable health promotion practices would further align the manuscript with current global public health priorities | We have included more information in the recommendation and discussion sections that places emphasis on adolescent empowerment and agency and how it can contribute to equity, inclusion and sustainable health promotion. For instance, information in the recommendation section now states:  “It is recommended based on our findings and supported by HPS framework, that schools as a critical environment in the development and change process of adolescents, should be a key component of promoting healthy lifestyles and environments in adolescents. as a relatively controlled and safe space for them. The results support and recommend initiatives in which adolescents are empowered to act as change agents within their school environment and broader social environments. By equipping adolescents with skills, confidence, and opportunities for leadership, these initiatives can foster meaningful change beyond the classroom, extending to families, peer groups, and communities. Furthermore, it will promote equity, inclusion, and sustainable health promotion practices. To more fully understand the impacts of such adolescent-engaged citizen science initiatives, it is recommended to evaluate the longer-term “ripple effects” that such programs can foster, including how adolescent-led change influences norms and practices in their immediate environments.  Fostering a more comprehensive life orientation curriculum in South African high schools that is embedded in the HPS framework for the holistic development of learners that encompasses subjects like physical education, life skills, health education, and career guidance can be a more positive experience for all and has the potential for inspiring lifelong learnings through motivating and engaging students in activities that are co-created with learners themselves (Line 529-544). |
| Minor Comment: The relatively small sample size should not be framed as a primary limitation of the study. From a qualitative and PAR perspective, the value of the research lies in transferability rather than statistical generalization. The authors rightly note that PAR approaches become increasingly difficult to manage with larger samples. Reframing this point more positively would help avoid applying quantitative evaluation criteria to a participatory qualitative design. | Thank you for your comment. We have revised the section title to “Strengths and limitations” and revised the sample size discussion positively. The strengths and limitation section now reads as:  “This study is the first Our Voice project that was completed with adolescents in Africa in which they participated in all the four stages of the OV method. Also, thematic analysis, advocacy and interventions were conducted with the adolescents to ensure a holistic perspective embedded in socioecological and HPS frameworks. Possible limitations identified are i) the heterogeneity in data collection across the schools. Interviews were conducted in only three of the schools with adolescents and their caregivers to support the data collected via the mobile application during the covid-pandemic. Also, although two mobile applications were used in data collection, both captured factors using comparable instructions and prompts. Additional variables (i.e. hygiene and safety) were introduced in three schools following preliminary data analysis from the first three schools, resulting in partial data availability for those indicators. However, all data were ultimately interpreted through thematic analysis workshops in which the CS collectively analysed and validated the results. ii) As the study was conducted in specific contexts, the findings may not be generalisable to other settings” (Line 546-557). |

| **Reviewer 2 Comments** | **Response to reviewer** |
| --- | --- |
| **Essential Revisions**  **1. Heterogeneity in Data Collection Across Schools**  **Location:** Methods (Lines 153–161) and Limitations (Lines 528–530)  The scope of data collection differed across schools:   - Only 3 schools collected home/neighbourhood data. - Hygiene and safety data were limited to low-SEA schools. - Two different mobile tools were used.   While partially acknowledged in Limitations, the implications for cross-SEA comparison are not explicitly stated.  Better to insert a clarifying sentence in the Methods for logic behind this difference in methodology (Section 2.3.2 or 2.4): | Thank you for the comment. We included more information regarding the reason behind the differences in the scope of data collection in the methods (Line 154--165).  “The mobile applications that were used for this study to collect data were the Discovery Tool, which is a neighbourhood environmental assessment mobile application developed by the Our Voice Global Citizen Science Research Initiative at Stanford University (29), and the Epicollect 5, developed by the Centre for Genomic Pathogen Surveillance as part of the Big Data Institute at Oxford University (30). We switched to the use of the Discovery Tool from the Epicollect 5 after data collection from 3 schools due to an established relationship with the Discovery Tool platform developer and technical support team, enabling real-time technical support. However, the instructions, prompts and usability of both tools was comparable. Both mobile applications can be used on either the Android or Apple iOS, and can collect data such as photos, GPS-based walking route mapping, and geo-tagged audio narratives/voice notes. Both tools have been used in a range of countries (31-34). The mobile applications do not require internet/data connection when being used; a Wi-Fi or internet connection is only needed when the data is being downloaded”  We have also included the differences as a possible limitation in the strengths and limitations section in Line 549-557.  “Possible limitations identified are i) the heterogeneity in data collection across the schools. Interviews were conducted in only three of the schools with adolescents and their caregivers to support the data collected via the mobile application during the covid-pandemic. Also, although two mobile applications were used in data collection, both captured factors using comparable instructions and prompts. Additional variables (i.e. hygiene and safety) were introduced in three schools following preliminary data analysis from the first three schools, resulting in partial data availability for those indicators. However, all data were ultimately interpreted through thematic analysis workshops in which the CS collectively analysed and validated the results. ii) As the study was conducted in specific contexts, the findings may not be generalisable to other settings” |
| **2. Qualitative Analysis Transparency**  **Location:** Lines 213–218  The description of thematic analysis is brief. It is unclear whether:   - An inductive approach was used, - Themes generated by citizen scientists were triangulated with researcher coding.   **Expand analysis description by 2–3 sentences stating type of qualitative analysis, theme generation and coding procedure.** | Thank you for your comment. We have included more description of the thematic analysis of the interviews by the researchers and the thematic analysis workshop with the citizen scientists in the methods section. The manuscript now reads:  “In a facilitated thematic analysis workshop, which was convened and facilitated by the research team, the CS were provided with de-identified photographs they had taken with the mobile device and associated verbatim narratives and themes from the interviews. The groups of CS were asked to sort through the various photographs, verbatim narratives and themes generated through researcher coding of the interviews. The CS validated themes and contributed to refining existing themes and generating additional ones (Fig 2). Once these themes had been identified, the groups were asked to prioritize those factors which they felt were barriers or facilitators and, more specifically, proffer feasible solutions to the barriers and suggest which types of changemakers could champion the change” (Line 187-194).    “All data collected using the mobile applications were downloaded to two central servers once the CS completed their discovery walks. Researchers de-identified photographs and associated narratives of data collected with the mobile applications and printed them out for analysis in addition to the themes generated from the interviews for further in-depth. Also, the interviews were inductively thematically analysed and themes printed out for further in-depth analysis by the CS in the first three set of schools. Only data collected by the adolescents and caregivers from each school were presented during the thematic analysis workshop. The CS validated researcher-coded themes and contributed to refining existing themes and generating additional ones. A detailed description of the analytic process of thematic workshop has been described earlier in the “Discuss and thematically analyse the findings” section (Line 231-239). |
| Location: Lines 534–537  The statement that lower socioeconomic status “is a significant contributor” may overstate findings from primarily qualitative data where “significance” in statistical terms cannot be measured.  Instead of “Lower socioeconomic stat… is a significant contributor…” write “findings suggest association of lower socio economic status with additional structural and resource related barriers” | Thank you for the comment. We have revised the statement as suggested to “findings suggest association of lower socio-economic status with additional structural and resource related barriers” (Line 559-560). |

| **Journal Requirements** | **Response to reviewer** |
| --- | --- |
| Please provide separate figure files in .tif or .eps format.  For more information about figure files please see our guidelines:  LINK  <https://journals.plos.org/globalpublichealth/s/figures>  <https://journals.plos.org/globalpublichealth/s/figures#loc-file-requirements> | We have separated the figures into separate files in .tif format as per the guidelines |
| . Please remove any photos of children (Figures 1,2,3,4 & 5) from your submission. | Thank you for the comment. We have reviewed Figures 1,2,3, 4 & 5 and removed photos of children that are identifiable, but we have kept some that are not identifiable.  For Figure 2, where the faces of the facilitator-researchers are visible, we have included signed PLOS Copyright Content Permission forms. The facilitators are also authors of this manuscript. The figure includes back views of adolescents; however, we can remove the photo if necessary. |
| Some material included in your submission may be copyrighted. According to PLOS’s copyright policy, authors who use figures or other material (e.g., graphics, clipart, maps) from another author or copyright holder must demonstrate or obtain permission to publish this material under the Creative Commons Attribution 4.0 International (CC BY 4.0) License used by PLOS journals. Please closely review the details of PLOS’s copyright requirements here: PLOS Licenses and Copyright. If you need to request permissions from a copyright holder, you may use PLOS's Copyright Content Permission form.  Please respond directly to this email or email the journal office and provide any known details concerning your material's license terms and permissions required for reuse, even if you have not yet obtained copyright permissions or are unsure of your material's copyright compatibility.  Potential Copyright Issues:  Figures 1,2,3,4,5,6 & 7 include an image of an identifiable person. Please provide written confirmation or release forms, signed by the subject(s) (or their parent/legally authorized guardian), giving permission to be photographed and to have their images published under our CC-BY 4.0 license.  Otherwise, we kindly request that you remove the photograph. | We have revised most of the figures containing pictures of identifiable children and replaced some of the pictures to include non-identifiable persons. For Figure 2, where the faces of the facilitator-researchers are visible, we have included signed PLOS Copyright Content Permission forms. The facilitators are also authors of this manuscript. The figure includes back views of adolescents; however, we can remove the photo if necessary.  We have total of 4 figures and not 7 in the revised manuscript namely  Figure 1: The Our Voice method 4-step process  Figure 2: Discuss (The thematic analysis workshop)  Figure 3: Discover (Samples of data collected by the citizen scientists)  Figure 4: Change (Infrastructure repairs) |
| Please amend your detailed online Financial Disclosure statement. This is published with the article. It must therefore be completed in full sentences and contain the exact wording you wish to be published.  a) State the initials, alongside each funding source, of each author to receive each grant. For example: “This work was supported by the National Institutes of Health (####### to AM; ###### to CJ) and the National Science Foundation (###### to AM).”  b) State what role the funders took in the study. If the funders had no role in your study, please state: “The funders had no role in study design, data collection and analysis, decision to publish, or preparation of the manuscript.” | We have amended our financial disclosure statement to reflect how it should appear in the final publication. We have included the initials of each author alongside the corresponding funding sources. We have also removed the statement from the manuscript, in accordance with the Instructions for Authors. |
| Please ensure that the funders and grant numbers match between the Financial Disclosure field and the Funding Information tab in your submission form. Note that the funders must be provided in the same order in both places as well. | The funders and grant numbers match between the Financial Disclosure field and the Funding Information tab in your submission form. |
| 3. We note your Data Availability statement as follows:  "All relevant data are included in the manuscript and as figures. The complete dataset for the "Sociodemographic characteristics and Body Mass Index (BMI) of adolescents in Cape Town categorised by their school socioeconomic area" during the current study are not publicly available due to ethical and privacy restrictions but may be made available from the corresponding author upon request"  In this instance it seems there may be acceptable restrictions in place that prevent the public sharing of your minimal data. However, in line with our goal of ensuring long-term data availability to all interested researchers, PLOS’ Data Policy states that authors cannot be the sole named individuals responsible for ensuring data access (<http://journals.plos.org/globalpublichealth/s/data-availability#loc-acceptable-data-sharing-methods>). | We have discussed this with our data team and can make accessible the sociodemographic characteristics and Body Mass Index (BMI) data of adolescents in Cape Town, categorised by their school socioeconomic area.  We have revised the Data Availability statement as:  “All relevant qualitative data are included within the manuscript and its figures. The quantitative dataset, *“*Sociodemographic characteristics and Body Mass Index (BMI) of adolescents in Cape Town categorized by their school socioeconomic area*,”* analysed during the current study, is available in the ZivaHub repository (University of Cape Town) at <https://doi.org/10.25375/uct.31982106>” |
