# Supplementary material for: Adolescent-led Our Voice method to promote healthy and active lifestyles and environments in South Africa
Source: PLOS Glob Public Health. 2026 Sep 2;6(9):e0007141. doi: 10.1371/journal.pgph.0007141 (PMC13537583; doi:10.1371/journal.pgph.0007141)
Supplement: Supplementary file 4 [file pgph.0007141.s005.docx]

June 6, 2026

Dear Editor,

Many thanks for the constructive comments you have sent us. We sincerely thank the reviewers for their careful reading of our manuscript and for providing constructive feedback and suggestions. We have considered each suggestion and have compiled our responses to all the comments.

Full details of our responses to the comments are below and tracked changes in the manuscript. The line numbers included in the table below align with the manuscript revision in tracked changes.

With best wishes,

Dr. Feyi Wayas and Co-authors

| **Reviewer 2 Comments** | **Response to reviewer** |
| --- | --- |
| **General Assessment**  The authors have made **substantial efforts to address prior reviewer comments**, particularly in clarifying methodology, expanding the discussion, and improving transparency regarding data collection heterogeneity. Most of the **claimed revisions (pp. 91–100)** are indeed implemented in the manuscript.  However, the revision remains **predominantly additive rather than analytical**. The manuscript continues to fall short in terms of **qualitative methodological rigor and depth of analysis**. | Thank you for the comment. We have included detailed responses to your itemised comments below. Most of the previous revision completed were meant to be additive as they provided more context and clarification to the introduction and methods. However, we have added more analytical context to the results and discussion in manuscript as detailed in the relevant sections below. We have also provided responses to the comments regarding the qualitative methodological rigor and depth of analysis in relevant sections below. |
| Insufficient Qualitative Rigor and Transparency  Issue  The manuscript describes thematic analysis but lacks key methodological components expected in qualitative research.  Specific Comments   - Lines 226–230 (Analysis section):   - Coding is described as being conducted by two researchers, but:     - No description of coding framework/codebook structure     - No mention of inter-coder agreement or reconciliation process     - No indication of data saturation - Provide:   - A brief coding framework or thematic structure   - Description of how disagreements were resolved   - Statement on whether thematic saturation was achieved | Thank you for your comment. Our research approach is qualitative participatory action research-based approach, supplemented with descriptive quantitative analysis. We described each step of the Our Voice method which included the Discover (data collected), Discuss (analyse and validate data), Advocate and Change. The terminology we may have used to describe the details of the DISCUSS (qualitative analysis) may not have adequately captured or clarified the process. Therefore, while the qualitative analysis process remains unchanged, we have more appropriately termed the analysis as a qualitative framework analysis approach guided by an adapted setting- based socioecological framework (Individual, Home, School and Neighbourhood Domains). Of note, the socioecological framework is not fixed and rigid but rather focuses on experiences and behaviours at multiple levels. The objective of the analysis was to systematically map barriers subcategories within the study’s socioecological domains categories. Consequently, findings are presented as subcategories within each domain category of the adapted framework. This approach enabled the identification of barriers across the setting domains while maintaining alignment with the study's conceptual framework.  All qualitative analysis i.e. the interviews, photos and transcribed information and the workshop data were analysed and presented using the study’s adapted socioecological framework. We have also redefined the facilitated thematic workshop as data validation and solutions workshop. With regards to saturation, the study utilized a predefined sample of between 10-15 adolescents in each school which in qualitative analysis is adequate for our methodological approach to support a rigorous analysis and generate meaningful insights relevant to the research questions.    We have included all these details in the manuscripts which now reads as:  Line 122- 125:” The study utilized a predefined sample of between 10-15 adolescents in each school which in qualitative research is adequate for our methodological approach to support a rigorous analysis and generate meaningful insights relevant to the research questions”  Line 190- 208: “A qualitative framework analysis approach (36) guided by an adapted setting- based socioecological framework (Individual, Home, School, and Neighbourhood Domains) was utilised for qualitative data analysis. A preliminary analysis of the interview was conducted by the researchers. Two researchers familiarised themselves with the interview transcripts. An analytical framework was developed based on the study’s adapted socioecological domains (individual, home, school, and neighbourhood) to organize the data. Findings were coded as subcategories within each domain category of the framework. Discrepancies in interpretation of codes were resolved through discussion and consensus among the coders and the research team.  In a researcher- facilitated data validation and solutions workshop in each school, the CS were provided with unanalysed de-identified photographs they had taken with the mobile device and associated verbatim narratives and the list of subcategories within the adapted socioecological framework categories from the interviews. The groups of CS were asked to sort through these data in groups or individually and then in a plenary, the CS validated the data and contributed to refining existing categories and generating additional ones, all of which were categorized according to the study’s socioecological framework (Fig 2). Also, in plenary, the CS proffered perceived feasible solutions to the barriers categorised and suggested relevant changemakers that could champion the change”. The final data categorisation and suggested solutions to the barriers were documented on a white board or flipchart during the workshop.  Line 302- 312: The validated data in each school, which was collected through a uniform data validation and solutions workshop process and included only findings that were explicitly validated or agreed upon by the citizen scientists during these workshops were subsequently compared by two researchers and grouped according to similarities and differences across schools that collected the same types of data. Diet and physical activity data were collected across all schools, enabling a direct comparison between "low" and "middle/high" SEA categories. For hygiene and safety data, which were identified as unique challenges for low -SEA schools, the analysis was focused on the later three low SEA schools. Discrepancies of comparison were resolved through discussion and consensus among research team. Suggested solutions were not compared across schools, as each school focused on barriers perceived to be actionable within its own context steps rather than produce directly comparable findings across settings. |
| - **Lines 188–193 (Thematic workshop):**   - Participant validation is mentioned but lacks detail. - Clarify:   - How participant validation influenced final themes   - Whether any themes were modified or rejected based on participant input   .  \ | Line 190- 208: “A qualitative framework analysis approach (36) guided by an adapted setting- based socioecological framework (Individual, Home, School, and Neighbourhood Domains) was utilised for qualitative data analysis. A preliminary analysis of the interview was conducted by the researchers. Two researchers familiarised themselves with the interview transcripts. An analytical framework was developed based on the study’s adapted socioecological domains (individual, home, school, and neighbourhood) to organize the data. Findings were coded as subcategories within each domain category of the framework. Discrepancies in interpretation of codes were resolved through discussion and consensus among the coders and the research team.  In a researcher- facilitated data validation and solutions workshop in each school, the CS were provided with unanalysed de-identified photographs they had taken with the mobile device and associated verbatim narratives and the list of subcategories within the adapted socioecological framework categories from the interviews. The groups of CS were asked to sort through these data in groups or individually and then in a plenary, the CS validated the data and contributed to refining existing categories and generating additional ones, all of which were categorized according to the study’s socioecological framework (Fig 2). Also, in plenary, the CS proffered perceived feasible solutions to the barriers categorised and suggested relevant changemakers that could champion the change”. The final data categorisation and suggested solutions to the barriers were documented on a white board or flipchart during the workshop. |
| Heterogeneity in Data Collection Not Analytically Addressed  Issue  Different schools contributed different types of data (e.g., interviews vs no interviews; additional domains introduced later).  Evidence   - Lines 149–151, 553–555 - Interviews conducted only in initial schools - Hygiene and safety introduced later - Major Action Required: - Either:   - Stratify findings by data availability (early vs later schools), OR - Explicitly justify why pooled analysis remains valid | We have provided evidence for the heterogeneity in the data collected as the reviewer indicated. Our justification for the pooled analysis of healthy eating and physical activity data across the six schools’ and safety and hygiene data for the later low -SEA schools are:  - A uniform citizen science validation and solutions workshop was conducted across all participating schools. This standardized approach allowed citizen scientists to review the data, provide new insights, and ensure that the process remained the same across different locations.  - The study, using a stringent data inclusion process, in included findings that were explicitly validated or agreed upon by the citizen scientists during these workshops.  - Comparability of Data: The study was designed as a descriptive/exploratory comparing similar barrier across schools that collected the same types of data. For example, diet and physical activity data were collected across all schools, enabling a direct comparison between "low" and "high" categories. For specific issues like hygiene and safety, which were identified as unique challenges for low Socio-Economic Areas (SEA), the pooled analysis was focused on the later three low SEA schools.  This information is provided in the manuscript in Line 267-276:  “The validated data in each school, which was collected through a uniform data validation and solutions workshop process and included only findings that were explicitly validated or agreed upon by the citizen scientists during these workshops were subsequently compared by two researchers and grouped according to similarities and differences across schools that collected the same types of data. Diet and physical activity data were collected across all schools, enabling a direct comparison between "low" and "middle/high" SEA categories. For hygiene and safety data, which were identified as unique challenges for low -SEA schools, the analysis was focused on the later three low SEA schools. Discrepancies of comparison were resolved through discussion and consensus among research team. Suggested solutions were not compared across schools, as each school focused on barriers perceived to be actionable within its own context steps”. |
| **Limited Analytical Depth of Results**  **Issue**  Results (Tables 3–6; Lines 289–374) are largely **descriptive listings** of barriers and solutions.  **Concerns**   - No hierarchy or prioritization of themes - No cross-domain synthesis - Limited interpretation of **why differences exist between SEAs** - Add:   - A short **analytical synthesis paragraph per domain**   - Explicit comparison between **low vs middle/high SEA**   - Identification of **dominant or recurring themes** | Thank you for the comments. We conducted comparisons of the barriers both across and within the SEA schools, as presented in Tables 3–6. We have provided additional details in the Methods section regarding the information presented in these tables. Furthermore, the Results and Discussion sections highlight the similarities and differences in the barriers identified across schools.  We did not conduct comparisons of the recommended solutions, as these were context-specific and were not intended for cross-site implementation comparisons. Our Voice” approach is participatory action research (PAR) rooted in citizen science principles. Its objective is to understand and improve a specific local context rather than produce directly comparable findings across settings. This is noted in Line 116: The expected outcomes will ultimately be to co-create strategies and solutions to address their barriers.  Lastly, we have expanded the Results section to provide more detail on the similarities and contrasts observed, which are further discussed in the Discussion section.  Details of the comparison conducted has been provided in the method, result and discussion sections in the manuscript. |
| **Socioecological Framework Not Operationalized**  **Issue**  Although the socioecological model is referenced (Lines 72–74, 226–227), it is **not used analytically**.   - Explicitly map findings across levels:   - Individual   - Interpersonal   - Institutional (school)   - Community - Consider adding a **summary figure or table** | For this study, we adapted a setting- based socioecological framework (Individual, Home, School and Neighbourhood Domains) which is not uncommon. This decision was guided by the nature of the data, as barriers and facilitators were predominantly described in relation to these environments rather than as individual-level factors. This approach is consistent with socioecological principles. It captures influences across multiple environmental contexts, while providing a structure that is more closely aligned with the data and study objectives. Tables 3-6 and our results are presented based on this adapted setting-based socioecological framework.  We do not consider a summary figure or table necessary, as the information is already presented in the results section. Including an additional figure or table would largely duplicate the findings and unnecessarily increase the length of the manuscript. |
| **. Overextension of Conclusions**  **Issue**  The manuscript claims effectiveness of the Our Voice method.  **Evidence**   - **Lines 44–46 (Abstract)** - **Lines 430–443 (Discussion)**   **Concern**   - Study is exploratory/pilot without comparative evaluation - **Major Action Required:** - Moderate claims:   - Replace “effective” with “feasible” or “promising”   - Clarify absence of causal inference | We have replaced “effective” with promising in the abstract and discussion (Line 45)  While participants reported that the project empowered and encouraged them to implement changes, the study design does not support causal inference. The qualitative approach was intended to explore participants' experiences, perceptions, and reported outcomes rather than to establish whether the project directly caused these changes.  With regards to the absence of causal inference, our study’s objective, similar to most qualitative research objective, was not to have casual inference. Our study was exploratory and designed to describe the implementation and outcomes of the of the Our Voice method) that was adolescent-led in promoting healthy and active lifestyles and environments. As such, we do not think it is necessary to clarify absence of casual inference as it is stated in our objective |
| . Methodological Justification for Tool Change   - Lines 154–160   - Tool switch is explained - Add 1–2 lines on:   - Potential impact on data comparability | We have explained the tool switch as indicated . There is minimal impact on data comparability as the prompt (questions) and instruction were similar and there was no intra school change of tool. As such, there is no expected significant data comparability concerns. We have included that in the manuscript as:  Line 160-161: “However, the instructions, prompts and usability of both tools was comparable, which is expected to have minimal impact for data comparability” |
| **Integration of Advocacy/Change Outcomes**   - **Lines 415–425**   **Issue:**   - Intervention outcomes are described narratively but not analyzed - Add brief interpretation:   - What enabled successful implementation?   - Differences across schools? | We appreciate the comment. Of note, our study was primarily descriptive and exploratory in nature, with the aim of documenting the process and outcomes of the Our Voice method rather than evaluating intervention effectiveness which is beyond the scope of this study.  Our objective was to describe how adolescents identified barriers and developed actionable solutions within their school contexts. Schools were encouraged to implement at least one feasible solution to an identified barrier, resulting in school-led interventions initiated by adolescents and approved by school authorities.  Importantly, suggested solutions were not systematically compared across schools. Although some barriers were similar, each school prioritized actions that were considered feasible and relevant within its local context and available resources. Consequently, the intervention implemented varied across schools according to the priorities identified by participants and the decisions of local stakeholders. We however indicated that “The buy-in and participation of adolescents and the school authority in the intervention to promote healthy lifestyles and environments is imperative” (Line 43-45).  We are unable to provide further explanations for differences in implemented solutions or outcomes across schools, as such comparative analyses of the change or suggested solutions were not part of the study objectives and fall beyond the scope of the present manuscript. |
| **Language and Clarity**   - Minor grammatical inconsistencies throughout - Example:   - **Line 28:** “Fostering adolescents’ agency and empowerment, can drive…” → remove comma | Thank you, we have checked and corrected grammatical inconsistencies in the manuscript. |
| **Redundancy in Discussion**   - **Lines 455–470**   - Repetition of known LMIC barriers | The section provided context to barriers that were identified as crosscutting in the schools and as such are not repetition but to reiterate that these barriers are still prevalent in schools and highlight proffered solutions that may also be relevant in other settings. |
| **Table Presentation**   - Tables 3–6:   - Very dense - Highlight **key themes** or summarizing in-text | We have revised the terminology for our qualitative analysis to “ qualitative framework analysis approach” guided by an adapted setting- based socioecological framework (Individual, Home, School and Neighbourhood Domains). We provided categories of the socioecological framework and subcategories within these domains. As such, we do not have key themes. However, we have reviewed and revised the table to check for grammatical correction and merged some subcategories but retained the information to maintain the integrity of the data validated during the data validation and solutions workshop . |
