# Supplementary material for: Adolescent-led Our Voice method to promote healthy and active lifestyles and environments in South Africa
Source: PLOS Glob Public Health. 2026 Sep 2;6(9):e0007141. doi: 10.1371/journal.pgph.0007141 (PMC13537583; doi:10.1371/journal.pgph.0007141)
Supplement: Peer review history

# Peer review: Decision Letter 0

**DOI:** 10.1371/journal.pgph.0007141.r001

**Authors:** Katrien Janin

19 Feb 2026

PGPH-D-25-03964

Adolescent-led Our Voice method to promote healthy and active lifestyles and environments in South Africa

PLOS Global Public Health

Dear Dr. Wayas,

Thank you for submitting your manuscript to PLOS Global Public Health. After careful consideration, we feel that it has merit but does not fully meet PLOS Global Public Health’s publication criteria as it currently stands. Therefore, we invite you to submit a revised version of the manuscript that addresses the points raised during the review process.

The manuscript has been evaluated by two reviewers, and their comments are available below.

The reviewers have raised a number of concerns that need attention. They request additional information on the context of your study, and suggest focusing on the qualitative PAR-based investigation part of your study. They also request that you include direct quotations in your study, either within the results narrative or as an additional column in the tables, to provide clear evidence that the reported themes genuinely emerged from the adolescents’ lived experiences,

Could you please carefully revise the manuscript to address all comments raised?

Please submit your revised manuscript by Apr 05 2026 11:59PM. If you will need more time than this to complete your revisions, please reply to this message or contact the journal office at globalpubhealth@plos.org. When you're ready to submit your revision, log on to https://www.editorialmanager.com/pgph/ and select the 'Submissions Needing Revision' folder to locate your manuscript file.

- A letter that responds to each point raised by the editor and reviewer(s). You should upload this letter as a separate file labeled 'Response to Reviewers'.
- A marked-up copy of your manuscript that highlights changes made to the original version. You should upload this as a separate file labeled 'Revised Manuscript with Track Changes'.
- An unmarked version of your revised paper without tracked changes. You should upload this as a separate file labeled 'Manuscript'.

Guidelines for resubmitting your figure files are available below the reviewer comments at the end of this letter.

We look forward to receiving your revised manuscript.

Kind regards,

Katrien G. Janin, PhD

Staff Editor

PLOS Global Public Health

Journal Requirements:

1. Please provide separate figure files in .tif or .eps format.

For more information about figure files please see our guidelines:  LINK

https://journals.plos.org/globalpublichealth/s/figures

https://journals.plos.org/globalpublichealth/s/figures#loc-file-requirements

2. Please remove any photos of children (Figures 1,2,3,4 & 5) from your submission.

3. Some material included in your submission may be copyrighted. According to PLOS’s copyright policy, authors who use figures or other material (e.g., graphics, clipart, maps) from another author or copyright holder must demonstrate or obtain permission to publish this material under the Creative Commons Attribution 4.0 International (CC BY 4.0) License used by PLOS journals. Please closely review the details of PLOS’s copyright requirements here: PLOS Licenses and Copyright. If you need to request permissions from a copyright holder, you may use PLOS's Copyright Content Permission form.

Please respond directly to this email or email the journal office and provide any known details concerning your material's license terms and permissions required for reuse, even if you have not yet obtained copyright permissions or are unsure of your material's copyright compatibility.

Potential Copyright Issues:

Figures 1,2,3,4,5,6 & 7 includes an image of an identifiable person. Please provide written confirmation or release forms, signed by the subject(s) (or their parent/legally authorized guardian), giving permission to be photographed and to have their images published under our CC-BY 4.0 license.

Otherwise, we kindly request that you remove the photograph.

If the reviewer comments include a recommendation to cite specific previously published works, please review and evaluate these publications to determine whether they are relevant and should be cited. There is no requirement to cite these works unless the editor has indicated otherwise.

Additional Editor Comments (if provided):

[Note: HTML markup is below. Please do not edit.]

Reviewers' comments:

Reviewer's Responses to Questions

**Comments to the Author**

1. Does this manuscript meet PLOS Global Public Health’s publication criteria?

Reviewer #1: Yes

Reviewer #2: Yes

**********

2. Has the statistical analysis been performed appropriately and rigorously?

Reviewer #1: N/A

Reviewer #2: Yes

**********

3. Have the authors made all data underlying the findings in their manuscript fully available (please refer to the Data Availability Statement at the start of the manuscript PDF file)?

The PLOS Data policy

Reviewer #1: No

Reviewer #2: Yes

**********

4. Is the manuscript presented in an intelligible fashion and written in standard English?

Reviewer #1: Yes

Reviewer #2: Yes

**********

Reviewer #1: The manuscript addresses a highly relevant and timely topic in global public health by examining an adolescent-led, school-based intervention aimed at promoting healthy and active lifestyles in a context that is often underrepresented in the literature.

The study is innovative in both its methodological approach and its ethical stance, particularly by amplifying adolescents’ voices in settings where young people’s perspectives are frequently marginalized. The decision to move beyond the identification of barriers and to explicitly advocate for change further strengthens the public health relevance of the work.

Despite these strong points, several recommendations and suggestions should be addressed to further strengthen the rigour, coherence, and conceptual positioning of the manuscript before it can be considered for publication in PLOS Global Public Health.

Major Comments:

Introduction:

The introduction section of the manuscript is brief and have room for improvement. It would benefit from more explicitly foregrounding Participatory Action Research (PAR) as a central theoretical and methodological pillar of the study. Although PAR is mentioned, its role is not sufficiently highlighted given that the Our Voice method is a well-established and consolidated participatory approach rather than merely a data collection tool. Expanding the introduction to better articulate the contribution of PAR to school-based health promotion would strengthen the conceptual framing of the study.

Relatedly, it is recommended that the authors situate their work more clearly within the existing literature on PAR by incorporating recent systematic reviews or meta-analyses that examine participatory and action-oriented methodologies in youth or school-based health promotion. In the same vein, given the strong use of photo-based evidence and environmental documentation, it would be appropriate to reference key studies on photo-elicitation methods (e.g., Photovoice), which align closely with the role attributed to adolescents as citizen scientists in this manuscript.

The manuscript also implicitly aligns with a salutogenic approach to health and with the principles of Health-Promoting Schools. Given the strong emphasis on agency, empowerment, and sustainability, the authors are encouraged to position their work more explicitly within this framework. Referencing key international bodies such as the World Health Organization or UNESCO would provide a stronger external theoretical anchor and reinforce the global relevance of the study. The advocate for change component, in particular, would benefit from being explicitly linked to salutogenic principles and adolescent agency.

Another distinctive strength of the study is its multi-component intervention design. This feature could be more clearly articulated in the introduction and supported with references to prior literature on multi-component school-based health promotion interventions. Doing so would help contextualise the intervention and clarify how the present study extends existing approaches.

Methods:

From my view, the manuscript may not need to be framed as a mixed-methods study. While the descriptive quantitative data are appropriate, the primary contribution of the article clearly lies in the qualitative analysis and in the participatory processes involving adolescent citizen scientists. The quantitative elements appear largely descriptive and do not substantially inform the interpretation of the findings. Presenting the study explicitly as a qualitative PAR-based investigation may therefore provide greater methodological coherence. Notably, the “Discover” phase, where evidence is quantified, contributes more to qualitative pattern identification than to inferential quantitative analysis.

Results:

Regarding the results, the credibility and transferability of the findings would be substantially strengthened by including verbatim excerpts from the adolescent participants. Integrating direct quotations, either within the results narrative or as an additional column in the tables, would provide clear evidence that the reported themes genuinely emerged from the adolescents’ lived experiences, rather than being solely the result of researcher interpretation. This addition would significantly enhance the trustworthiness of the qualitative analysis and is considered one of the most important areas for improvement.

Discussion:

The manuscript would benefit from moving beyond a predominantly socio-ecological interpretation toward a more explicit salutogenic framing. The strong emphasis on advocacy, sustainability, and student agency offers an excellent opportunity to articulate how adolescent-led participatory interventions contribute to the creation of health-promoting school environments over time. Making this conceptual shift more explicit would strengthen the theoretical contribution of the study.

Finally, in the recommendations section, greater emphasis could be placed on adolescent empowerment and the development of agency. Highlighting how this approach contributes to equity, inclusion, and sustainable health promotion practices would further align the manuscript with current global public health priorities.

Minor Comment:

The relatively small sample size should not be framed as a primary limitation of the study. From a qualitative and PAR perspective, the value of the research lies in transferability rather than statistical generalization. The authors rightly note that PAR approaches become increasingly difficult to manage with larger samples. Reframing this point more positively would help avoid applying quantitative evaluation criteria to a participatory qualitative design.

Reviewer #2: The write up needs minor revisions and clarifications like

1. Heterogeneity in Data Collection Across Schools

2. Qualitative Analysis Transparency

3. Cross-SEA Interpretation

details of each point are given in the attached file

**********

what does this mean?). If published, this will include your full peer review and any attached files.

**Do you want your identity to be public for this peer review?**  If you choose “no”, your identity will remain anonymous but your review may still be made public.

For information about this choice, including consent withdrawal, please see our Privacy Policy

Reviewer #1: No

Reviewer #2: No

**********

[NOTE: If reviewer comments were submitted as an attachment file, they will be attached to this email and accessible via the submission site. Please log into your account, locate the manuscript record, and check for the action link "View Attachments". If this link does not appear, there are no attachment files.]

Attachment Submitted filename: plos global review.docx

# Author comment: Author response to Decision Letter 1

**DOI:** 10.1371/journal.pgph.0007141.r002

14 Apr 2026

Attachment Submitted filename: Response to reviwer_ PLOS v3.docx

# Peer review: Decision Letter 1

**DOI:** 10.1371/journal.pgph.0007141.r003

**Authors:** Katherine Kokkinias

10 May 2026

PGPH-D-25-03964R1

Adolescent-led Our Voice method to promote healthy and active lifestyles and environments in South Africa

PLOS Global Public Health

Dear Dr. Wayas,

Thank you for submitting your manuscript to PLOS Global Public Health. After careful consideration, we feel that it has merit but does not fully meet PLOS Global Public Health’s publication criteria as it currently stands. Therefore, we invite you to submit a revised version of the manuscript that addresses the points raised during the review process.

The manuscript has been reassessed by two reviewers, and their comments are available below.

The reviewers have raised additional concerns that need attention. Overall, the reviewers would like additional information to improve methodological transparency. They would like additional details about data saturation, intercoder agreement metrics or how disagreements between coder were handled. They would like further justification for pooling data based on heterogeneity of the schools. They also suggest tempering conclusion statement regarding the effectiveness of the Our Voice method.

Could you please revise the manuscript to carefully address the concerns raised?

Please submit your revised manuscript by Jul 07 2026 11:59PM. If you will need more time than this to complete your revisions, please reply to this message or contact the journal office at globalpubhealth@plos.org. When you're ready to submit your revision, log on to https://www.editorialmanager.com/pgph/ and select the 'Submissions Needing Revision' folder to locate your manuscript file.

Please include the following items when submitting your revised manuscript:

- A letter that responds to each point raised by the editor and reviewer(s). You should upload this letter as a separate file labeled 'Response to Reviewers'.
- A marked-up copy of your manuscript that highlights changes made to the original version. You should upload this as a separate file labeled 'Revised Manuscript with Track Changes'.
- An unmarked version of your revised paper without tracked changes. You should upload this as a separate file labeled 'Manuscript'.

Guidelines for resubmitting your figure files are available below the reviewer comments at the end of this letter.

As the corresponding author, your ORCID iD is verified in the submission system and will appear in the published article. PLOS supports the use of ORCID, and we encourage all coauthors to register for an ORCID iD and use it as well. Please encourage your coauthors to verify their ORCID iD within the submission system before final acceptance, as unverified ORCID iDs will not appear in the published article. *Only*  the individual author can complete the verification step; PLOS staff *cannot*  verify ORCID iDs on behalf of authors.

We look forward to receiving your revised manuscript.

Kind regards,

Katherine Demi Kokkinias, Ph.D.

Staff Editor

PLOS Global Public Health

Journal Requirements:

If the reviewer comments include a recommendation to cite specific previously published works, please review and evaluate these publications to determine whether they are relevant and should be cited. There is no requirement to cite these works unless the editor has indicated otherwise.

Additional Editor Comments (if provided):

[Note: HTML markup is below. Please do not edit.]

Reviewers' comments:

Reviewer's Responses to Questions

**Comments to the Author**

Reviewer #1: All comments have been addressed

Reviewer #2: (No Response)

**********

publication criteria?

Reviewer #1: Yes

Reviewer #2: Partly

**********

3. Has the statistical analysis been performed appropriately and rigorously?

Reviewer #1: N/A

Reviewer #2: No

**********

4. Have the authors made all data underlying the findings in their manuscript fully available (please refer to the Data Availability Statement at the start of the manuscript PDF file)?

The PLOS Data policy

Reviewer #1: Yes

Reviewer #2: Yes

**********

5. Is the manuscript presented in an intelligible fashion and written in standard English?

Reviewer #1: Yes

Reviewer #2: Yes

**********

Reviewer #1: I would like to congratulate the authors for the rigorous and extensive work undertaken to improve the manuscript. The revisions carried out have substantially strengthened the quality, clarity, and overall coherence of the paper.

In particular, the recommendations and suggestions previously raised by this reviewer have been appropriately addressed. Consequently, I consider that the manuscript, in its current form, could be recommended for publication.

Reviewer #2: The authors have made substantial efforts to address prior reviewer comments, particularly in clarifying methodology, expanding the discussion, and improving transparency regarding data collection heterogeneity. Most of the claimed revisions (pp. 91–100) are indeed implemented in the manuscript.

However, the revision remains predominantly additive rather than analytical. The manuscript continues to fall short in terms of qualitative methodological rigor and depth of analysis.

Specific Comments are mentioned in detail in the attached file

**********

what does this mean?). If published, this will include your full peer review and any attached files.

**Do you want your identity to be public for this peer review?**  If you choose “no”, your identity will remain anonymous but your review may still be made public.

For information about this choice, including consent withdrawal, please see our Privacy Policy

Reviewer #1: No

Reviewer #2: No

**********

[NOTE: If reviewer comments were submitted as an attachment file, they will be attached to this email and accessible via the submission site. Please log into your account, locate the manuscript record, and check for the action link "View Attachments". If this link does not appear, there are no attachment files.]

Attachment Submitted filename: plos global review 2.docx

# Author comment: Author response to Decision Letter 2

**DOI:** 10.1371/journal.pgph.0007141.r004

30 Jun 2026

Attachment Submitted filename: Response to reviewer_ PLOS_2nd reviewerdocx.docx

# Peer review: Decision Letter 2

**DOI:** 10.1371/journal.pgph.0007141.r005

**Authors:** Julia Robinson

4 Aug 2026

Adolescent-led Our Voice method to promote healthy and active lifestyles and environments in South Africa

PGPH-D-25-03964R2

Dear Dr Wayas,

We are pleased to inform you that your manuscript 'Adolescent-led Our Voice method to promote healthy and active lifestyles and environments in South Africa' has been provisionally accepted for publication in PLOS Global Public Health.

Before your manuscript can be formally accepted you will need to complete some formatting changes, which you will receive in a follow up email. A member of our team will be in touch with a set of requests.

Please note that your manuscript will not be scheduled for publication until you have made the required changes, so a swift response is appreciated.

IMPORTANT: The editorial review process is now complete. PLOS will only permit corrections to spelling, formatting or significant scientific errors from this point onwards. Requests for major changes, or any which affect the scientific understanding of your work, will cause delays to the publication date of your manuscript.

If your institution or institutions have a press office, please notify them about your upcoming paper to help maximize its impact. If they'll be preparing press materials, please inform our press team as soon as possible -- no later than 48 hours after receiving the formal acceptance. Your manuscript will remain under strict press embargo until 2 pm Eastern Time on the date of publication. For more information, please contact globalpubhealth@plos.org.

Thank you again for supporting Open Access publishing; we are looking forward to publishing your work in PLOS Global Public Health.

Best regards,

Julia Robinson

Executive Editor

PLOS Global Public Health

***********************************************************

Reviewer Comments (if any, and for reference):

Reviewer's Responses to Questions

**Comments to the Author**

Reviewer #1: All comments have been addressed

**********

publication criteria?

Reviewer #1: Yes

**********

3. Has the statistical analysis been performed appropriately and rigorously?

Reviewer #1: Yes

**********

4. Have the authors made all data underlying the findings in their manuscript fully available (please refer to the Data Availability Statement at the start of the manuscript PDF file)?

The PLOS Data policy

Reviewer #1: Yes

**********

5. Is the manuscript presented in an intelligible fashion and written in standard English?

Reviewer #1: Yes

**********

Reviewer #1: The authors have satisfactorily addressed all my comments and recommendations. I therefore recommend the manuscript for publication

**********

what does this mean?). If published, this will include your full peer review and any attached files.

**Do you want your identity to be public for this peer review?**  If you choose “no”, your identity will remain anonymous but your review may still be made public.

For information about this choice, including consent withdrawal, please see our Privacy Policy

Reviewer #1: No

**********
